# Distinct ultrastructural phenotypes of glial and neuronal alpha-synuclein inclusions in multiple system atrophy

**DOI:** 10.1093/brain/awae137

**Published:** 2024-05-02

**Authors:** Carolin Böing, Marta Di Fabrizio, Domenic Burger, John G J M Bol, Evelien Huisman, Annemieke J M Rozemuller, Wilma D J van de Berg, Henning Stahlberg, Amanda J Lewis

**Affiliations:** C-CINA, Biozentrum, University of Basel, Basel 4058, Switzerland; Laboratory of Biological Electron Microscopy, Institute of Physics, School of Basic Sciences, Ecole Polytechnique Federale Lausanne, Lausanne, Vaud 1015, Switzerland; Department of Fundamental Microbiology, Faculty of Biology and Medicine, University of Lausanne, Lausanne, Vaud 1015, Switzerland; Laboratory of Biological Electron Microscopy, Institute of Physics, School of Basic Sciences, Ecole Polytechnique Federale Lausanne, Lausanne, Vaud 1015, Switzerland; Department of Fundamental Microbiology, Faculty of Biology and Medicine, University of Lausanne, Lausanne, Vaud 1015, Switzerland; Department of Anatomy and Neurosciences, section Clinical Neuroanatomy and Biobanking, Amsterdam Neuroscience, Amsterdam University Medical Centre, Vrije University Amsterdam, Amsterdam 1081 HZ, The Netherlands; Department of Anatomy and Neurosciences, section Clinical Neuroanatomy and Biobanking, Amsterdam Neuroscience, Amsterdam University Medical Centre, Vrije University Amsterdam, Amsterdam 1081 HZ, The Netherlands; Department of Pathology, Amsterdam Neuroscience, Amsterdam University Medical Centre, Vrije University Amsterdam, Amsterdam 1081 HZ, The Netherlands; Amsterdam Neuroscience program Neurodegeneration, Amsterdam University Medical Centre, Vrije University Amsterdam, Amsterdam 1081 HZ, The Netherlands; Department of Anatomy and Neurosciences, section Clinical Neuroanatomy and Biobanking, Amsterdam Neuroscience, Amsterdam University Medical Centre, Vrije University Amsterdam, Amsterdam 1081 HZ, The Netherlands; Amsterdam Neuroscience program Neurodegeneration, Amsterdam University Medical Centre, Vrije University Amsterdam, Amsterdam 1081 HZ, The Netherlands; Laboratory of Biological Electron Microscopy, Institute of Physics, School of Basic Sciences, Ecole Polytechnique Federale Lausanne, Lausanne, Vaud 1015, Switzerland; Department of Fundamental Microbiology, Faculty of Biology and Medicine, University of Lausanne, Lausanne, Vaud 1015, Switzerland; Laboratory of Biological Electron Microscopy, Institute of Physics, School of Basic Sciences, Ecole Polytechnique Federale Lausanne, Lausanne, Vaud 1015, Switzerland; Department of Fundamental Microbiology, Faculty of Biology and Medicine, University of Lausanne, Lausanne, Vaud 1015, Switzerland

**Keywords:** multiple system atrophy, correlative light and electron microscopy, disease pathology, post-mortem human brain, alpha-synuclein

## Abstract

Multiple system atrophy is characterized pathologically by the accumulation of alpha-synuclein (aSyn) into glial cytoplasmic inclusions (GCIs). The mechanism underlying the formation of GCIs is not well understood.

In this study, correlative light and electron microscopy was employed to investigate aSyn pathology in the substantia nigra and putamen of post-mortem multiple system atrophy brain donors.

Three distinct types of aSyn immuno-positive inclusions were identified in oligodendrocytes, neurons and dark cells presumed to be dark microglia. Oligodendrocytes contained fibrillar GCIs that were consistently enriched with lysosomes and peroxisomes, supporting the involvement of the autophagy pathway in aSyn aggregation in multiple system atrophy. Neuronal cytoplasmic inclusions exhibited ultrastructural heterogeneity resembling both fibrillar and membranous inclusions, linking multiple systems atrophy and Parkinson’s disease. The novel aSyn pathology identified in the dark cells, displayed GCI-like fibrils or non-GCI-like ultrastructures suggesting various stages of aSyn accumulation in these cells.

The observation of GCI-like fibrils within dark cells suggests these cells may be an important contributor to the origin or spread of pathological aSyn in multiple system atrophy. Our results suggest a complex interplay between multiple cell types that may underlie the formation of aSyn pathology in multiple system atrophy brain and highlight the need for further investigation into cell-specific disease pathologies in multiple system atrophy.

## Teaser

Using advanced imaging techniques to examine brain tissue from people who died from multiple system atrophy, Böing *et al.* find that different cell types display distinct types of alpha-synuclein accumulations. The results highlight the complex interplay between multiple cell types that may underlie the disease pathogenesis.

## Introduction

Multiple system atrophy is part of a spectrum of neurodegenerative movement disorders, including Parkinson’s disease and dementia with Lewy bodies, characterized by the progressive accumulation of the protein alpha-synuclein (aSyn) into pathological inclusions in susceptible regions of the brain.^1-4^ The predominant accumulation of aSyn in oligodendrocytes as glial cytoplasmic inclusions (GCIs) is specific to multiple system atrophy and distinguishes it neuropathologically from other synucleinopathies where aSyn inclusions are predominantly neuronal.^4-12^ Since aSyn is abundantly expressed in neurons^13-15^ and has only low expression levels in oligodendrocytes,^16-19^ the source and abundance of accumulated aSyn in oligodendrocytes is puzzling.

The leading hypothesis for the formation of GCIs is through the intercellular transmission of a pathological form of aSyn from neurons to oligodendrocytes.^20,21^ This hypothesis is supported by the fact that neuronal aSyn aggregates can be found at early disease stages and present a progressive pattern of pathology, with aggregates growing in dimensions and numbers with disease duration.^22-24^ The mechanism for the intercellular transmission is thought to be caused by aSyn fibrils spreading through the brain via a prion-like mechanism.^25-27^ In accordance with this hypothesis, various experiments have found that aSyn seeds originating from multiple system atrophy brain are more potent in spreading aSyn pathology in injection models of rat brain when compared to those derived from Parkinson’s disease or dementia with Lewy bodies brain.^28,29^ Moreover, recent structural studies have found that fibrils derived from the brain material of multiple system atrophy patients after sarkosyl detergent solubilization adopt unique structural conformations with distinct seeding properties when compared to Parkinson’s disease and dementia with Lewy bodies derived fibrils.^29-31^ These experiments have led to the hypothesis that the specific type of fibril strain dictates the differences in the clinical and pathological manifestations of the different aSyn diseases.^32^

However, the prion model in the context of aSyn spreading is rather controversial due to unanswered questions regarding its transmission from cell to cell.^33^ For instance, although it has been shown that aSyn can spread from one cell to another,^34-40^ multiple system atrophy brain lysates do not induce the same oligodendroglial pathology in animal models or cell cultures as observed in multiple system atrophy patient brains.^25,26,40-44^ Moreover, it remains unknown what causes the abnormal inclusion formation in the first place and whether it might be a consequence of a change in cellular environment or subcellular architecture. For example, it has been found in multiple system atrophy oligodendrocytes that autophagy and iron metabolism are perturbed, which may either be a consequence of, or a prerequisite for, inclusion formation.^45^

We recently showed that the majority of aSyn-positive aggregates in the Parkinson’s disease brain are primarily composed of accumulated membrane fragments and cellular organelles rather than fibrils,^46^ adding to the uncertainty surrounding the pathomechanism of synucleinopathies. With existing animal and cell models not yet being able to simulate the formation of human multiple system atrophy disease pathology, new insights must come from the study of post-mortem human brain. In this study, we used correlative light and electron microscopy (CLEM) to establish the aSyn pathology-structure relationship in different cell types in post-mortem human multiple system atrophy brains. We document that autophagy organelles are consistently enriched within fibrillar oligodendrocytic pathology and that neuronal inclusions in multiple system atrophy can consist of densely packed vesicles and membranes without the presence of fibrils. Finally, we describe the presence of GCI-like fibrils in aSyn immuno-positive microglia for the first time. Our results highlight the structural differences in aSyn inclusions across cell types.

## Materials and methods

### Human post-mortem brain samples

We included eight multiple system atrophy-Parkinson’s variant brain donors (Donors A–H), who participated in the brain donation program from the Netherlands Brain Bank (www.brainbank.nl) with a post-mortem delay of <6 h (Supplementary Table 1). Brain regions were dissected at autopsy according to the standardized procedure of the NBB. Tissue blocks from the substantia nigra (SN) and putamen were fixed in 4% formalin for multiple system atrophy Donors A–C, E and F. Tissue from Donors D, G and H were fixed in in 2% paraformaldehyde and 2.5% glutaraldehyde in 0.15 M cacodylate buffer, supplemented with 2 mM calcium chloride, pH 7.4 for 24 h. Tissue from Donors A–C, E and F were post-fixed in 0.1% glutaraldehyde for 24 h before processing for EM.

All donors provided written informed consent for a brain autopsy and the use of the material and clinical information for research purposes. Detailed neuropathological and clinical information was made available, in compliance with local ethical and legal guidelines, and all protocols were approved by Vrije University Medical Center institutional review board. Demographic features and clinical symptoms were abstracted from the clinical files, including sex, age at symptom onset, age at death, disease duration, presence of dementia, core and supportive clinical features for multiple system atrophy.^47,48^

For pathological diagnosis, 7-μm-thick formalin-fixed paraffin-embedded sections were immuno-stained using antibodies against αSyn (clone KM51, 1:500, Monosan Xtr), amyloid-β (clone 4G8, 1:8000, Biolegend) and phosphorylated tau (p-tau, clone AT8, 1:500, ThermoFisher Scientific), as previously described.^49^ Braak and McKeith αSyn stages were determined using the BrainNet Europe (BNE) criteria.^50^ Based on Thal amyloid-β phases scored on the medial temporal lobe,^51^ Braak neurofibrillary stages^50^ and Consortium to Establish a Registry for Alzheimer’s Disease (CERAD) neuritic plaque scores,^52^ levels of Alzheimer’s disease (Ad) pathology were determined according to National Institute on Aging-Alzheimer's Association (NIA-AA) consensus criteria.^53^ Additionally, Thal cerebral amyloid angiopathy (CAA) stages,^54^ presence of ageing-related tau astrogliopathy (ARTAG),^55^ microvascular lesions and hippocampal sclerosis were assessed.

### Correlative light and electron microscopy

CLEM was performed as described previously.^46^ Briefly, 60-μm-thick tissue sections prepared with a vibratome (Leica VT1200) were collected and post-fixed in 2% osmium tetroxide reduced with 3% potassium ferrocyanide. Sections were then immersed in filtered thiocarbohydrazide and fixed again in 2% osmium tetroxide. After overnight staining in 1% uranyl acetate, sections were stained with lead aspartate, pH 5.5 at 60°C, dehydrated in a graded ethanol series and embedded in Durcopan resin. Hardened resin samples were trimmed and mounted on resin support blocks. Serial sections of 80–200 nm were cut using an ultramicrotome and alternatingly collected on electron microscopy grids and glass slides, respectively. Glass slides were processed for immunohistochemistry using antibodies against aSyn and immuno-positive aggregates detected by light microscopy at ×400 or ×630 magnification. Features in the tissue that were identifiable in both the light and electron microscopy images were used to guide the collection of EM images of the aSyn immuno-positive pathology. Donors A–D and F–H were used for CLEM.

To correlate specific cell types in the tissue, we performed immunolabelling on 15–40 μm free-floating brain sections and used fluorescence microscopy to map the positions of precursor and mature oligodendrocytes, microglia, astrocytes, neurons and aSyn immuno-positive neuronal inclusions in the sections. The sections were incubated with primary antibody overnight at 4°C and visualized with Alexa-conjugated secondary antibodies and DAPI (Biolegend #422801; 1/800 dilution) to label cell nuclei after incubation at room-temperature for 30 min. Antibodies used for fluorescence CLEM are listed in Supplementary Table 2. The sections were washed three times in 1× Tris-buffered saline (TBS) and mounted on glass slides in 50% TBS-glycerol for fluorescent imaging. *Z*-stacks were taken at 300 nm intervals over large areas in the tissue on a Leica Thunder Tissue imager, and deconvoluted using the integrated Thunder algorithm. The sections were then resin-embedded, and the imaged regions excised by laser-capture microdissection (Leica LMD7; 5× objective, laser power-60, aperture 1, speed- 5, specimen balance-0, pulse frequency 3500). CLEM sectioning was performed as described above. Adjacent slides were stained with toluidine blue (1% with 1% borax in H_2_0) for 1–2 min at 90°C and coverslipped for imaging on an Olympus VS200 slide scanner using a 40× oil objective.

### Immunohistochemistry

The sections on the glass slides were etched in a saturated potassium ethoxide solution for 3 min followed by washing in phosphate buffered saline (PBS). Antigen retrieval was carried out for Donors A–C, E and F with 100% formic acid for 10 min followed by steaming in Tris-EDTA, pH 9 for 30 min at 100°C. Endogenous peroxidases were quenched with 1% hydrogen peroxide in 10% methanol, before blocking in Dako REAL antibody diluent (Agilent). The sections were incubated in primary aSyn antibody solution for 4 h at room temperature (Antibody Clone 42, BD Biosciences; 1/100 dilution in buffer solution; Donors A–C, E and F) or 1 h at 37°C (Antibody LB509, ThermoFisher; 1/500 dilution in buffer solution; Donors D, G and H), before washing in PBS supplemented with 0.25% Triton X and incubation in secondary antibody (ImmPRESS Reagent Anti-Mouse Ig, Vector Laboratories) for 30 min at room temperature. Bound antibody complexes were detected using the permanent horseradish peroxidase (HRP) Green Kit (Zytomed Systems) with incubation for 3 min at room temperature. Sections were counterstained with haematoxylin, dehydrated and mounted on glass coverslips for imaging. Two different antibodies were used for the detection of aSyn pathology due to the prolonged duration of formalin fixation for Donors A–C, E and F, resulting in different antigenic properties of this tissue compared to Donors D, G and H.

### Fluorescent image correlation

The BigWarp plugin in FIJI (ImageJ Fiji, National Institutes of Health, USA; https://imagej.nih.gov/ij/) was used to correlate the fluorescence and toluidine blue images. The toluidine blue images from each CLEM cycle were aligned to each other to create a 3D stack, and maximum projections of the corresponding fluorescent image was warped onto the stack based on distinctive tissue features. For this rough alignment easy to recognize neuromelanin or large blood vessels were used as initial landmarks. Clearly correlating cell nuclei were added as landmarks in an iterative fashion until a fine alignment was sufficiently achieved to correctly correlate the cell of interest with confidence. Neurons, mature and precursor oligodendrocytes, astrocytes, and microglia were identified by their positive fluorescent signal, correlated to the correct position on the toluidine blue sections and imaged by transmission electron microscopy (TEM). The dark cells were easily identified from their distinctive morphology and staining pattern on the toluidine blue sections and confirmed by TEM imaging. Immunohistochemistry on adjacent slides, described above, was used to identify the aSyn immuno-positive dark cells. After the BigWarp fine alignment, the positions of the dark cells on the toluidine blue images were then back-correlated to the fluorescent images to identify any cell type-specific fluorescent staining.

### Immunogold labelling

Immunogold labelling was performed on the EM grids produced by CLEM. The resin was etched for 10 min in 1% periodic acid (Sigma), followed by three times washing each in water and washing buffer (BSA-c, Aurion; diluted 1/50) before blocking for 10 min in blocking solution (Aurion; diluted 1/5). The grids were incubated in primary antibody (IBA1, FUJIFILM Wako Pure Chemical Corporation; 1/25 dilution), or washing buffer for the no-primary antibody control, at room temperature for 60 min before washing six times in blocking solution. The grids were incubated with 10 nm protein A conjugated gold beads (Aurion) for 90 min at room temperature before washing three times each in TBS and water before being recontrasted with 1% uranyl acetate for 10 min. Ten individual EM montages were recorded each for aSyn immuno-positive dark cells (as localized by CLEM), aSyn immuno-negative dark cells, oligodendrocytes, and neurons compared to a non-primary antibody control. The micrographs were collected on a CM100 Biotwin (Philips) operated at 80 kV with a Lab6 filament and bottom mount TVIPS F416 camera at a pixel size of 1.8 nm/pixel. Gold beads were detected using the semi-automated convolutional neural network protocol of EMAN2^56^ followed by counting using the particle analysis tool in FIJI^57^ with the particle detection size between 12 and 100 pixels^2^ and a circularity cut-off between 0.8 and 1.0 (Supplementary Table 3; gold). The area of each cell type was calculated in FIJI^57^ by overlaying a grid of cross-hairs spaced at 300 000 nm^2^ for each image, and manual counting of the cross-hairs which fell inside or outside the cell (Supplementary Table 3; points). Quantification of the labelling density per cell type was calculated and preferential labelling was identified upon meeting the criteria that the % chi-squared value was over 10% of the total chi-squared value and, the relative labelling index was above 1 (Supplementary Table 4).^58^

### Multi-label immunofluorescence and confocal microscopy for detection of organelles in GCIs

To study the presence and co-localization of peroxisomes and lysosomes in GCIs, we performed a multi-label immunofluorescence of catalase (EP1929Y, Abcam ab76024) LIMP2 (LIMPII/SR-B2, Novus Biologicals NB400-129) and alpha-synuclein (LB509, Abcam ab27766) on 6 µm sections of adjacent formalin-fixed paraffin-embedded tissue blocks of the midbrain containing the SN. Briefly, the sections were deparaffinized, immersed in 10 mM citrate buffer pH 6.0, and heated to 100°C in a steam cooker for 30 min for antigen retrieval. First, the primary antibody against LIMP2 was diluted in TBS with Triton 0.1% (pH 7.4) and section were incubated for 90 min at room temperature. LIMP2 was detected and visualized with Envision (anti-rabbit) and tyramide A555 (A-K079; dilution 1:100 in Tris-HCl + 0.005% H_2_O_2_). Next, the sections were heated again using a steam cooker for 10 min to remove the LIMP2 primary antibody. After washing with TBS and 3% normal donkey serum in TBS + 0.1% Triton, the sections were incubated with catalase (dilution 1/400) and LB509 (dilution 1/200). The catalase and LB509 were visualized using donkey anti-rabbit A594 (A-S174; ThermoFisher) and donkey anti-mouse (A488+ A-S172; ThermoFisher), respectively. After counterstaining with DAPI (Sigma-Aldrich, 1/1000), the sections were mounted with Mowiol (Sigma-Aldrich) plus anti-fading agent DABCO.

### Imaging

Light microscopy images of selected glass slides containing aSyn immuno-positive structures were collected using a Leica Thunder microscope equipped with a DMI8 colour camera. The entire section was imaged in overlapping tiles at ×400 or ×630 (oil immersion) magnifications, and image tiles were merged into a single image using the LAS X software (Leica Microsystems).

TEM images of electron microscopy grids consecutive to those imaged by light microscopy were collected at room temperature on a 120 kV Tecnai G2 Spirit TEM microscope operated at 80 kV with a LaB6 filament and a side mounted EMSIS Veleta camera, a CM100 Biotwin (Philips) operated at 100 kV or a Tecnai Spirit BioTwin (FEI) operated at 120 kV with Lab6 filaments and bottom mounted TVIPS F416 cameras.

Light microscopy images were corrected for colour-blind readers by replacing the red channel with magenta, and both light and electron microscopy images were adjusted for brightness and contrast where necessary using FIJI.^57^

Confocal imaging was performed with a Leica TCS SP8 (Leica Microsystems) using an HC PL PAO CS2 100× oil objective lens, NA 1.40 and a pixel size of 30–50 nm. Sections were sequentially scanned for each fluorochrome with a pulsed white light laser at different wavelengths (DAPI: 405 nm; Alexa 488: 499 nm; Alexa 555: 565 nm; Alexa 594: 598 nm). All signals were detected using gated hybrid detectors in counting mode. *Z*-stacks (*z* = 6 μm; 1024 × 1024 pixels) were taken in the SN of multiple system atrophy Donor E. After scanning, the images were deconvoluted using CMLE algorithms in Huygens Professional (Scientific Volume Imaging, the Netherlands; https://svi.nl/Huygens-Professional), and their maximum projections (ImageJ, Fiji) were used to represent graphically the structures of interests and their morphologies. Final figures were created using Adobe Illustrator (CS6, Adobe Systems incorporated).

### Tomography

Tomograms were collected with a pixel size of ∼0.5 nm on a Jeol 2100 Plus at 200 kV equipped with a LaB6 filament and TVIPS camera, or a Talos F200C (ThermoFisher) operating at 200 kV equipped with an extreme field emission gun (X-FEG) electron source and a Ceta camera. Exposures of 0.5 s were collected every 2° from −60° to +60°. Tomograms were binned by a factor of 2 and filtered using a non-local means filter in Amira version 2021.2 (ThermoFisher Scientific). Segmentation of the fibrils and the lysosome crystal was carried out using the EMAN2 semi-automated convolutional neural network protocol^56^ and refined using the UCSF Chimera package^59^ and Amira. Membranes were segmented both semi-automatically with EMAN2 or manually using the b-spline tool in Amira. The fibril thickness distribution was extracted using the Amira Thickness Map module. The extracted data were thresholded to match the EMAN2 segmentation and to exclude the filament overlap when crossing each other.

## Results

**Figure 1:**
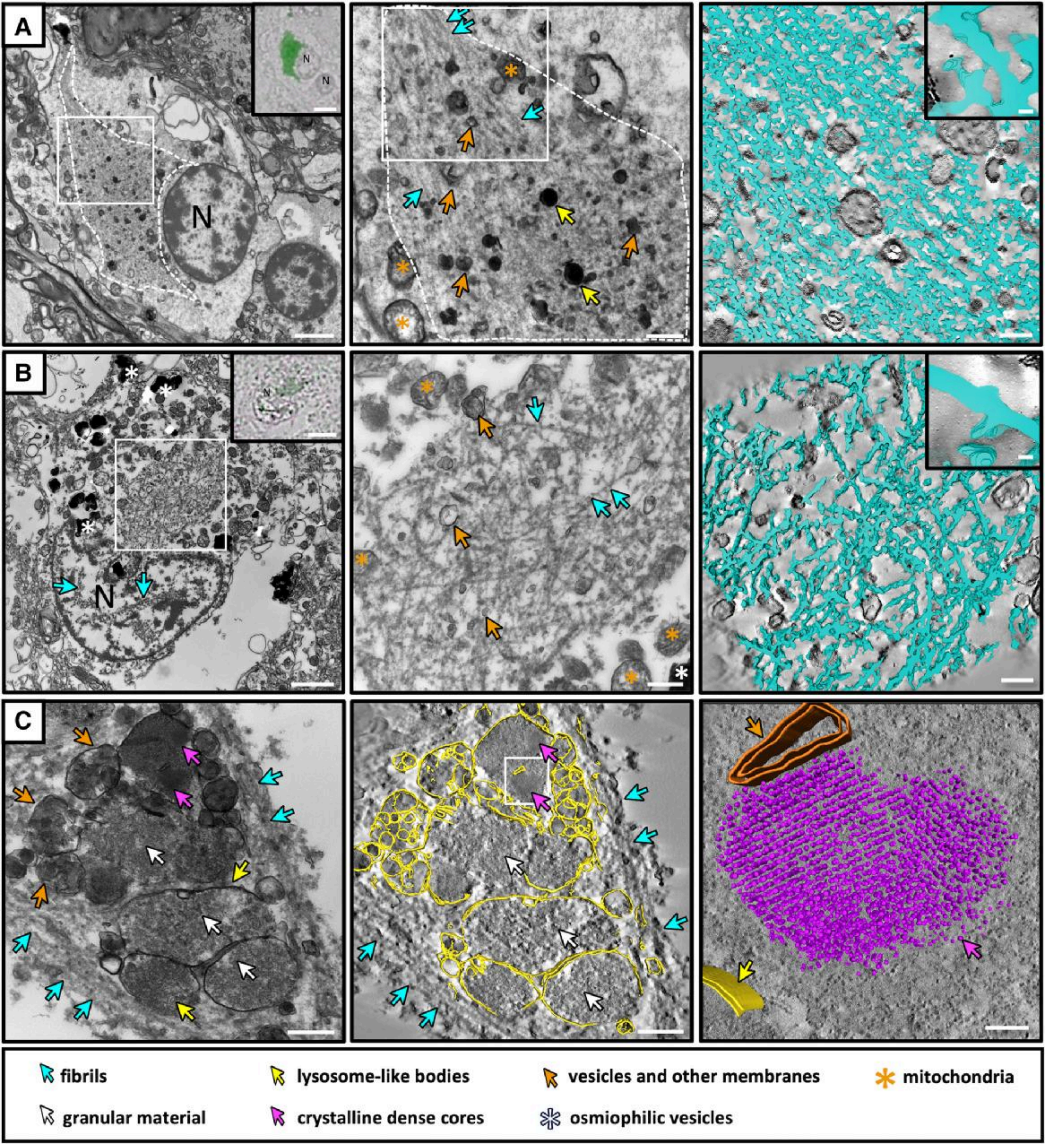
**Glial cytoplasmic inclusion fibrils co-localize with vesicles, lysosome-like bodies and peroxisomes.** Transmission electron microscopy (TEM) micrographs (*left* and *middle*) and segmentations of tomograms (*right*) showing different glial cytoplasmic inclusion (GCI) features localized by CLEM. Light microscopy images of alpha-synuclein (aSyn) immuno-staining used to identify GCIs is shown in the *inset*. (**A**) A GCI from the substantia nigra of Donor D. Osmiophilic vesicles, lysosome-like bodies and many vesicles can be seen amongst the long, unbranched and linearly arranged fibrils. (**B**) A GCI from the putamen of Donor C. Various vesicles and mitochondria can be seen amongst the fibrils, and nuclear fibrils are evident in some cases. The segmentation for both **A** and **B** (*right*) shows the long, linear arrangement of the fibrils in the GCI with an average width of 21 nm. (**C**) A large cluster of vesicles, lysosome-like bodies, and peroxisomes within a GCI from the putamen of Donor C (also shown in Supplementary Fig. 3b, *left*). The segmentation shows the position of the various membranous bodies (*middle*) and the crystalline structure typical of peroxisomes (*right*). N = nucleus. Scale bars: TEM, **A** and **B**, *left* = 2 µm, *middle* and *right* = 500 nm; EM, **C**, *left* and *middle* = 200 nm, *right* = 100 nm; light microscopy, *insets* = 5 µm.

**Figure 2:**
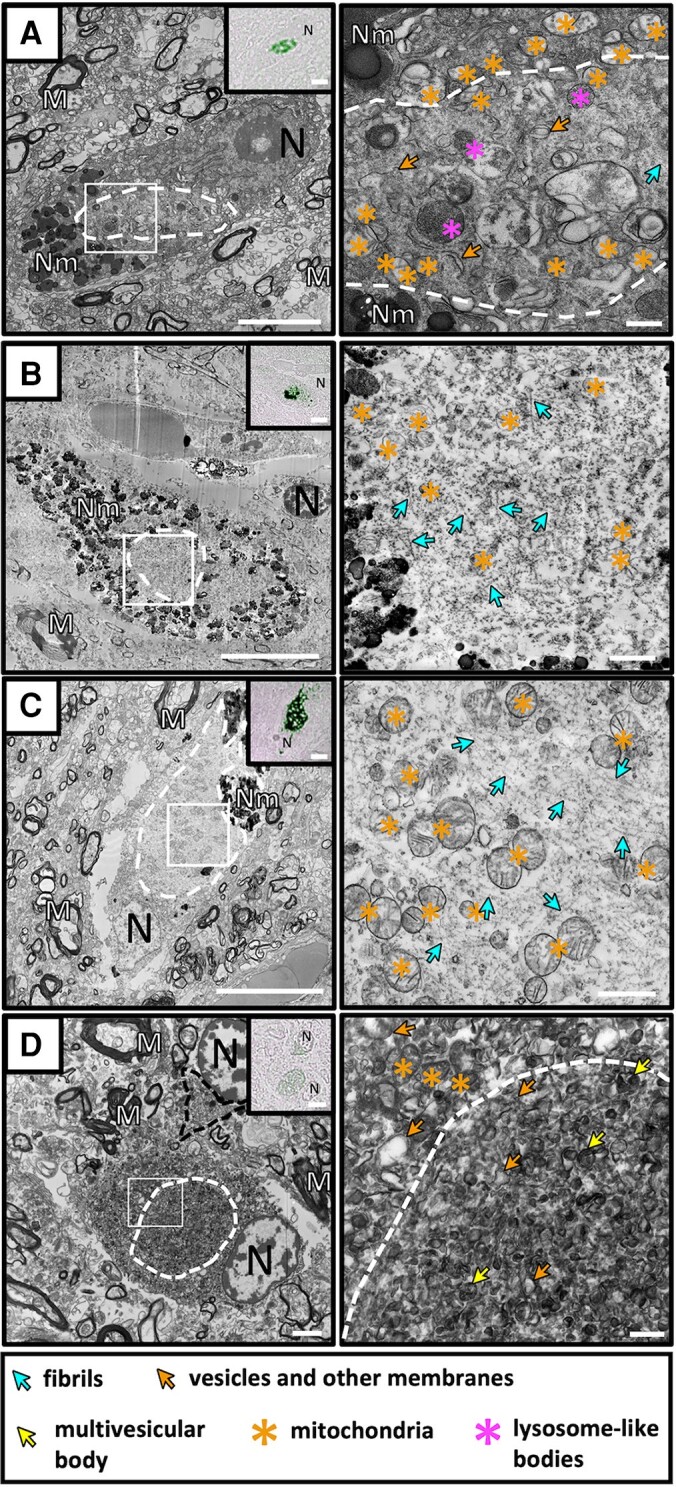
**Distinctive ultrastructures for neuronal alpha-synuclein-positive inclusions.** (**A**) Transmission electron microscopy (TEM) micrograph of a neuronal inclusion (white dotted line) localized within the substantia nigra (SN) of Donor D. A higher magnification of the white frame seen in **A** is shown. The interior of this inclusion consisted of a mixture of fibrils, vesicles, mitochondria, and multi-vesicular bodies. (**B**) TEM micrograph of a fibrillar neuronal inclusion from the SN of Donor F is shown. The fibrils are intermixed with mitochondria. (**C**) TEM micrograph of a fibrillar neuronal inclusion from the SN of Donor D where the mitochondria are clustered together. (**D**) TEM micrograph of a membranous neuronal inclusion (white dotted line) localized within the SN of Donor C (also shown in Fig. 5B). The globular neuronal inclusion was ultrastructurally distinct from a neighbouring fibrillar glial cytoplasmic inclusion (GCI; black dotted line). A higher magnification of the white frame seen in **A** is shown. The interior of the neuronal inclusion consisted of highly condensed lysosome-like bodies, vesicles and other membranous material. The cytoplasm surrounding the alpha-synuclein (aSyn) immuno-positive area consists of more condensed membranes and vesicles, including mitochondria. No fibrils could be observed. Light microscopy images showing aSyn immuno-positive staining are shown in the insets. N = nucleus. M = myelin. Nm = neuromelanin. Scale bars: EM low magnification = 2 µm, high magnification = 500 nm; light microscopy = 5 µm.

**Figure 3:**
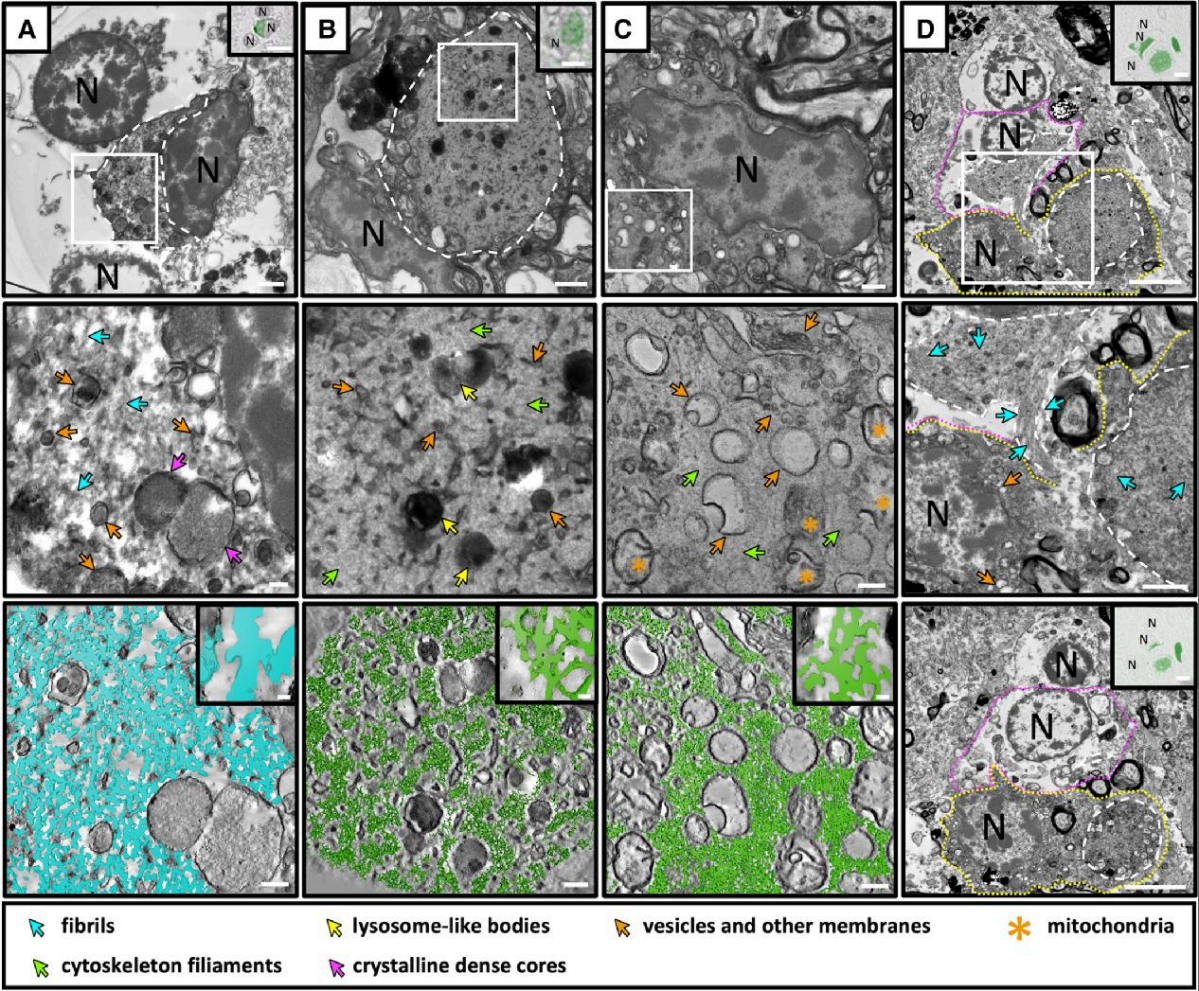
**Variable ultrastructures of dark cells suggest different cellular states of alpha-synuclein pathology.** Transmission electron microscopy (TEM) micrographs (*left* and *middle*) and segmented tomograms (*right*) of alpha-synuclein (aSyn) immuno-positive microglia. Light microscopy (LM) images of aSyn immuno-staining used to identify dark cells are shown in insets. (**A**) An aSyn immuno-positive microglia from the putamen of Donor C has an ultrastructure visually similar to glial cytoplasmic inclusions (GCIs) with long, unbranched and linearly arranged 22 ± 7 nm fibrils interspersed with vesicles, lysosome-like bodies and peroxisomes. (**B**) An aSyn immuno-positive dark cell consisting of highly branched 6 ± 3 nm filaments arranged in a high-density mesh across the cytoplasm of the cell. The filaments are interspersed with vesicles, lysosome-like bodies, and membrane fragments. Mitochondria can be seen bordering the inclusion. The filamentous mesh is identical to the ultrastructure making up the cytoplasm of the non-pathological dark cell (**C**), therefore, it most likely represents the cytoskeleton of the cell. This dark cell was localized in the surrounding cellular area to **B** and identified based on its morphology by EM alone. As it was immuno-negative for aSyn, no LM staining is shown. (**D**) *Left*: An immuno-positive dark cell with fibrillar ultrastructure (yellow dotted line) is adjacent to an immuno-positive oligodendrocyte (purple dotted line) containing a GCI. Immuno-positive areas are outlined with a white dashed line. *Middle*: A higher magnification of the image shown in **D** shows a patch of fibrils from the GCI extending into the dark cell. *Right*: A TEM micrograph of the same area on an adjacent grid, approximately 1.5 µm away in *z*-height, shows that the aSyn immuno-positive area of the dark cell is in the same cell as the nucleus identified in the image on the *left*. The GCI of the adjacent oligodendrocyte was no longer visible in this section. N = nucleus. Scale bars: EM = 2 µm (*left*), 200 nm (*middle* and *right*); LM = 5 µm.

To identify the ultrastructure of glial and neuronal aSyn inclusions in the multiple system atrophy brain, we used our previously described workflow of correlative light and electron microscopy.^46^ We collected high-quality human brain tissue of donors diagnosed with the Parkinson’s variant of multiple system atrophy (Supplementary Table 1), processed 15–60-μm-thick sections for electron microscopy using *en bloc* staining protocols and localized glial and neuronal inclusions using an antibody against aSyn. We localized 196 aSyn immuno-positive inclusions within the SN and putamen of seven multiple system atrophy brain donors (Supplementary Table 3). Based on the size, shape and heterochromatin pattern of the cell nuclei, as well as their local proximity to myelin sheaths, we identified 128 GCIs within six brain donors (Fig. 1 and Supplementary Figs 1–4), 20 neuronal cytoplasmic inclusions (NCIs) within five brain donors (Fig. 2 and Supplementary Figs 5–7), three axonal aSyn inclusions within three brain donors (Supplementary Fig. 8) and 47 dark cell cytoplasmic inclusions within five brain donors (dark cells; Figs 3 and 5 and Supplementary Figs 9, 10 and 16).

### Fibrillar glial cytoplasmic inclusions co-localize with autophagic organelles

We observed that GCIs in both the putamen and SN are composed of long, unbranched and linearly arranged fibrillar bundles that are highly decorated with varying degrees of amorphous proteinaceous material, cellular vesicles and organelles including mitochondria (Fig. 1A and B and Supplementary Figs 1–4), consistent with previous EM observations.^6,7,60-66^ The width of the fibril core, without the fuzzy coat, was an average of 21 nm ± 6 nm (Supplementary Fig. 11). No obvious ultrastructural differences were observed between the GCIs in the two brain regions, except that heterogenous dense material was observed to surround GCIs more frequently in the putamen (Supplementary Figs 3 and 4) compared to the SN (Supplementary Figs 1 and 2). This material is consistent with iron deposition and has also been proposed to be derived from degenerating myelin sheaths to which the cell is attached.^67^ However, we observed that many of the cell nuclei from the oligodendrocytes localized in the putamen were often misshapen or deformed (Supplementary Figs 3 and 4) compared to those in the SN (Supplementary Figs 1 and 2).

We additionally observed electron dense bodies co-localizing with the fibrils in 121 out of the 128 GCIs. The contents of these bodies contained different features such as smaller vesicles, membrane fragments, granular material and crystalline cores (Fig. 1C and Supplementary Figs 1 and 2). As these contents are consistent with the ultrastructure of lysosomes,^68-70^ autophagosomes or multi-vesicular bodies,^69^ and peroxisomes^71^ respectively, we performed confocal laser scanning microscopy on paraffin sections in adjacent tissue blocks from the same patient tissues. We consistently observed the clustering of lysosomal and peroxisome markers in GCIs confirming the cellular identity of these electron dense bodies. (Supplementary Fig. 12).

In a subset of cells containing GCIs we additionally observed the presence of fibrils inside the cell nucleus (Fig. 1B and Supplementary Fig. 13) consistent with previous observations.^2,72^

### Neuronal cytoplasmic inclusions have both fibrillar and membranous type ultrastructures

We observed that many of the NCIs in both the putamen and SN contained fibrillar material as previously reported; however, in our data the fibrils were always intermixed with varying degrees of vesicles, membrane fragments multivesicular bodies, lysosome-like bodies and mitochondria (Fig. 2A–C and Supplementary Figs 5–7). In some cases, the mitochondria were clustered around the periphery of the inclusions, consistent with other recent observations in Parkinson’s disease brain (Fig. 2B).^46^ In another example, the mitochondria in the inclusion were clearly clustered together amongst the fibrils, in an arrangement that has been previously attributed to the morphology of pale bodies in Parkinson’s disease (Fig. 2C).^73^

**Figure 4:**
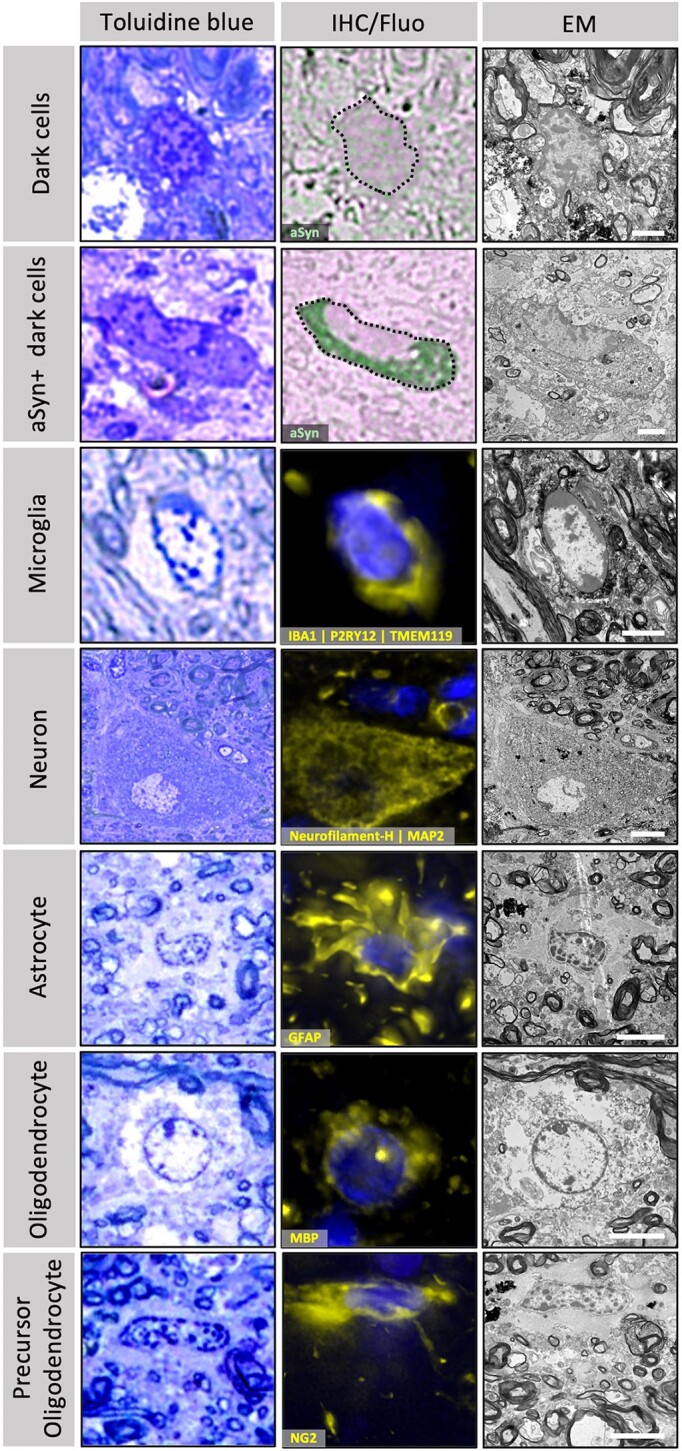
**Correlative light and electron microscopy showing toluidine blue staining, immuno-fluorescence or immunohistochemistry staining, and the electron micrograph of the same cell.** Toluidine blue staining highlights that dark cells are phenotypically distinct from other cell types. The dark cells were identified by the distinct phenotype of their nuclear toluidine blue staining, where both the heterochromatin and nucleoplasm are stained. No differences in the toluidine blue staining were observed between the dark cells and the alpha-synuclein (aSyn) immuno-positive dark cells, which were exclusively identified by immunohistochemistry (IHC) staining on adjacent sections (black dotted outline). For the other cell types, toluidine blue stained only the heterochromatin, leaving a clear nucleoplasm. Their specific cell type was identified by their positive staining for markers against neurons (neurofilament-H and MAP2 antibody cocktail), microglia (IBA1, P2RY12 and TMEM119 antibody cocktail), astrocytes (GFAP antibody), oligodendrocytes (MBP antibody) or precursor oligodendrocytes (NG2 antibody). Fluorescent images are maximum projections of *z*-stacks imaged in 30 µm free-floating brain sections. Toluidine blue, IHC and electron microscopy images were obtained from correlated 200 and 80 nm (respectively) ultrathin sections collected after resin embedding and correlative light and electron microscopy (CLEM) sectioning. The neuron shown here is also shown at lower magnification in Supplementary Fig. 15. Scale bars: dark cells and microglia = 2 µm; neuron = 10 µm; astrocyte, oligodendrocyte and precursor oligodendrocyte = 5 µm.

**Figure 5:**
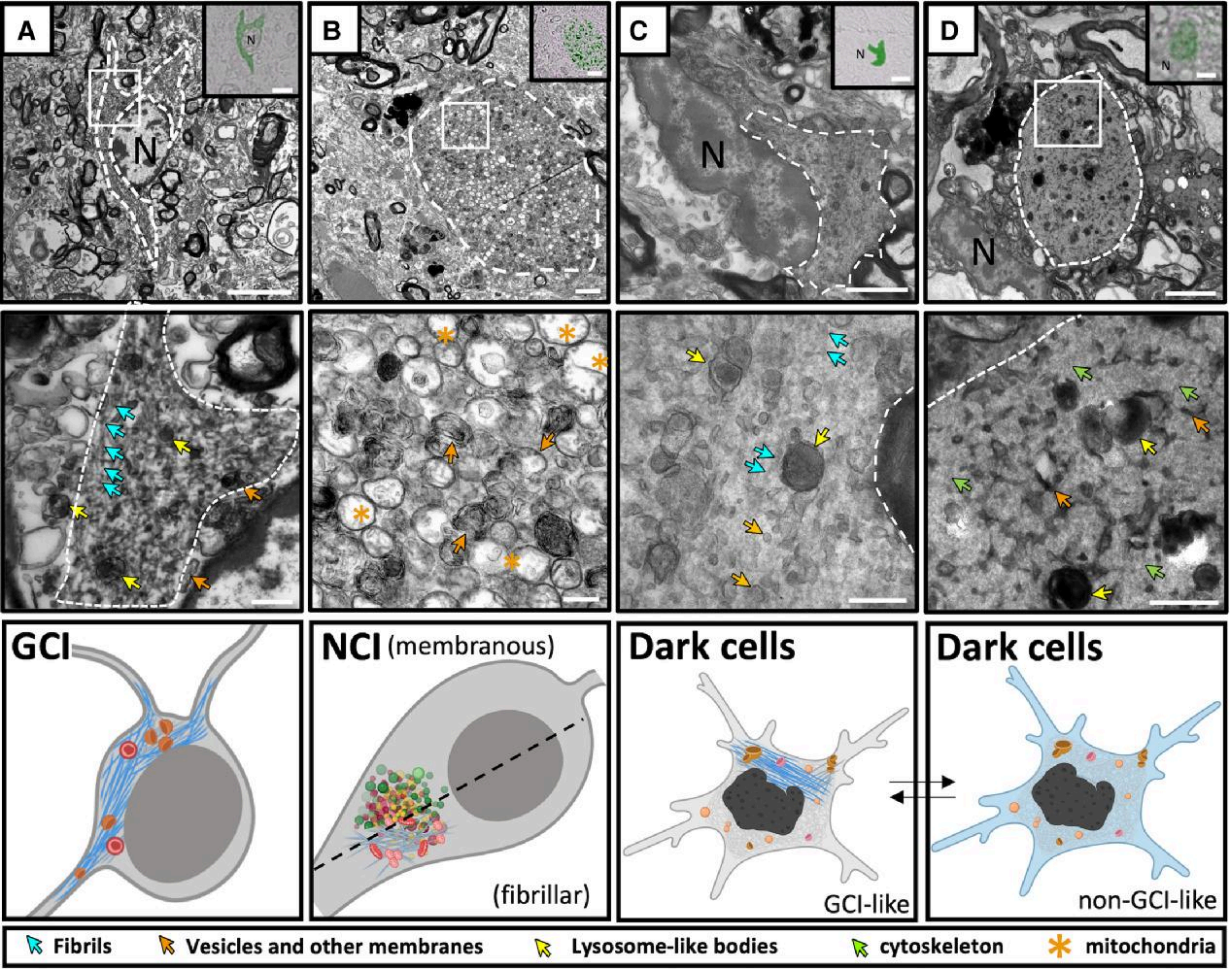
**Comparison of glial cytoplasmic inclusions, neuronal cytoplasmic inclusions and dark cell ultrastructures.** Transmission electron microscopy (TEM) micrographs (*left* and *middle*) and graphical representation (*right*) showing the ultrastructural composition of a glial cytoplasmic inclusion (GCI), neuronal cytoplasmic inclusion (NCI) and dark cell (white dotted lines) localized by correlative light and electron microscopy (CLEM). Light microscopy (LM) images of alpha-synuclein (aSyn) immuno-positive staining used to localize inclusions are shown in inserts. (**A**) GCIs are composed of long, linear fibrils which co-localize with vesicles, peroxisomes and lysosome-like bodies. The fibrils in this GCI extend into the cell’s processes. (**B**) NCIs can consist of densely packed vesicles and membrane fragments where no fibrils are visible, or can have a fibrillar ultrastructure intermixed with vesicles, membranes and mitochondria. (**C**) Dark cells can contain fibrillar bundles similar to a GCI, or non-fibrillar aSyn accumulation (**D**). These ultrastructures may represent different stages of aSyn accumulation in microglia. This figure was partially made with BioRender.com N = nucleus. Scale bars: EM = 2 µm (*left*), 500 nm (*middle*); LM = 5 µm.

We also observed two NCIs from the SN of two different donors that consisted of a highly dense accumulation of membrane fragments and vesicles (Figs 2D and 4B). Fibrils could not be identified within these inclusions; however, it is possible that their presence was obscured by the high density of vesicles. In one example, the membranous NCI was in close proximity to a GCI (Fig. 2D). No mitochondria was observed within the inclusion itself, in contrast to the neuronal inclusions within Parkinson’s disease brain.^46^ In this cell, mitochondria were found in the cytoplasm surrounding the aSyn immuno-positive inclusion, which was also highly enriched in densely accumulated vesicles of various sizes and mitochondria so that the immuno-positive area would have been virtually indistinguishable from the rest of the cytoplasm without the use of CLEM. Mitochondria were observed in the second membranous NCI (Fig. 5B) however were severely damaged as indicated by the loss of their internal cristae. In a subset of cells containing NCIs from the putamen and SN, we also observed the presence of fibrils inside the cell nucleus (Supplementary Figs 5E and F and 7B–E).

In addition to the NCIs, we observed three aSyn immuno-positive inclusions within cross-sections of myelinated axons (Supplementary Fig. 8). The ultrastructure of the aSyn inclusions within these axons showed accumulated fibrils intermixed with some small vesicles and mitochondria. There were no obvious lysosomes or autophagosomes present within the immuno-positive area. The accumulated fibrils were visually identical to the fibrils within GCIs, and their disordered arrangement distinguished them from the highly ordered cytoskeleton at the periphery of the axons.

### Fibrillar and non-fibrillar alpha-synuclein pathology identified in dark cells

aSyn immunoreactivity was identified in 47 cells that had noticeably misshapen cell nuclei with a condensed size and a distinctive heterochromatin pattern in the nucleoplasm giving them a dark appearance. Additionally, the cytoplasm of these cells was highly electron dense and the entire cytoplasm was aSyn immuno-positive (Figs 3, 5B and C and Supplementary Figs 9 and 10). In two examples, the immuno-positive dark cells were next to an immuno-negative oligodendrocyte showing a large, oval-shaped nucleus and sparsely populated cytoplasm, highlighting the morphological difference between the cell types (Supplementary Figs 9K and 10J).

The majority of the dark cells found in the putamen and SN of five separate patients showed an ultrastructure similar to that of GCIs and where the cytoplasm contained fibrillar bundles intermixed with enlarged lysosome-like bodies and peroxisomes (Figs 3A and 5C and Supplementary Figs 9 and 10). By tomography and segmentation, the width of the fibrils within these dark cells measured 22 ± 7 nm, similar to the width of the fibrils in GCIs (Supplementary Fig. 11).

A subset of the dark cells were found, where no fibrillar material was apparent, but the cytoplasm instead consisted of a highly dense proteinaceous material intermixed with membrane fragments, vesicles and lysosome-like bodies (Figs 3B and 5D and Supplementary Figs 9 and 10). Electron tomography subsequently revealed the proteinaceous material to be a highly branched network of filaments (Fig. 3B) with a width of 7 nm ± 3 nm (Supplementary Fig. 11). Membrane fragments and vesicles were intermixed within the filaments, with in-tact mitochondria observed at the periphery (Figs 3A and 5C and Supplementary Fig. 6). A comparison of this ultrastructure with that of an aSyn immuno-negative dark cell revealed the same branched 7 nm filamentous network within the cytoplasm (Fig. 3C and Supplementary Fig. 11). As the filament width and branched nature are consistent with the description of actin filaments, which were also observed in other non-aSyn-positive cells, it is likely that the thin filaments seen in the dark cells were the normal cytoskeleton of the cell, and not aSyn fibrils. However, it is also possible that thin (7 nm) aSyn fibrils are intermixed with the cytoskeletal filaments, as we would not be able to distinguish between the two with the resolution limitations of room-temperature electron microscopy in resin-embedded tissue.

In one notable example, we observed a fibrillar dark cell adjacent to an oligodendrocyte containing a GCI (Fig. 3D). There was no obvious cell membrane between the two cells, and there was a patch of fibrils connecting the fibrils of the GCI with the tissue in the aSyn immuno-positive dark cells, so that it appeared that the fibrils were being transferred from one cell type to the other. In this image the aSyn immuno-positive area of the dark cell was separated from the nearby nucleus by a region of sparse tissue showing some empty resin, raising some doubt about whether the nucleus belonged to the aSyn immuno-positive area, or if it was a separate cell. Therefore, we imaged the same region in the adjacent grids (approximately 1.5 μm away in the *z*-height) and observed that the cytoplasm of the dark cells in this section was densely packed over the entire region connecting the nucleus to the immuno-positive area confirming that they belonged to the same cell (Fig. 5D, right). Therefore, it is possible that this image represents evidence of mature fibrils spreading between cell types.

### Alpha-synuclein positive dark cells are not defined by common cell type-specific protein markers

Cells with a similar morphological description have been recently termed as ‘dark microglia’, as the distinctive morphological appearance of these cells by electron microscopy differentiates them from typical microglia and other cell types.^74-77^ Therefore, we attempted to confirm that the novel aSyn immuno-positive dark cells we identified were also of microglial origin. We first performed immunogold labelling on the imaged EM grids containing aSyn immuno-positive dark cells against the common microglial marker IBA1 (Supplementary Fig. 14 and Supplementary Table 3). We compared the labelling density of IBA1 between aSyn immuno-positive dark cells, and aSyn immuno-negative dark cells, oligodendrocytes and neurons, to a non-primary antibody control. The aSyn immuno-positive dark cells had been previously localized by CLEM and the aSyn immuno-negative cells were identified by their morphology in the surrounding areas on the same grids. 10 TEM montages were taken for each cell type and the number of gold beads counted within the boundary of each cell, as well as the area of the cell determined by the number of crosshairs (points) of a superimposed grid falling within the cell-boundary (Supplementary Fig. 14A and B and Supplementary Table 3). The number of gold beads varied from ∼17 to 1700 per cell for the IBA1 immuno-gold labelled grids (Supplementary Fig. 14C and Supplementary Table 3) and <10 for the non-primary antibody control (data not shown) indicating IBA1 binding specificity. Gold beads falling on empty resin were excluded from the analysis, as were areas containing neuromelanin granules as the beads were difficult to detect reliably against the dark background.

The average labelling density of IBA1 for each cell type was calculated as gold beads/point (Supplementary Fig. 14D). Dark cells showed the highest labelling density compared to the other cell types; however, the standard deviations overlapped significantly due to the large variability in the number of gold beads counted per image. A one-tailed, paired student *t*-test between the dark cells and the other cell types revealed that the differences in the labelling density between dark cells, oligodendrocytes and neurons was not significant (*P* > 0.05). However, the student *t*-test of dark cells compared to aSyn immuno-positive dark cells gave a *P*-value of < 0.05 indicating that the labelling density of aSyn immuno-negative dark cells was significantly higher than for aSyn immuno-positive dark cells.

To determine if IBA1 preferentially labelled dark cells over the other cell types, the expected distribution of gold particles was calculated and compared to the observed distribution of gold particles to determine the relative labelling index of each of the four cell types (Supplementary Table 4). Dark cells and oligodendrocytes showed a relative labelling index value >1 (Supplementary Fig. 14E), indicating preferential (non-random) labelling for IBA1, whereas aSyn immuno-positive dark cells and neurons showed a relative labelling index ≤ 1, indicating random labelling is occurring for these cell types (Supplementary Fig. 14E, left, and Supplementary Table 4). The statistical significance of differences between the observed and expected gold distribution was calculated by the two-sample chi-squared test with 3 degrees of freedom. The corresponding *P*-value was <0.05 for all cell types indicating that the observed and expected gold distribution is significantly different. Dark cells showed the largest contribution to the total chi-squared value, indicating that this labelling is more likely to contain a specific gold signal (Supplementary Fig. 14E right and Supplementary Table 4).

Preferential labelling of IBA1 was determined based on the satisfaction of two criteria being that the relative labelling index was >1, and the corresponding partial chi-squared value accounted for a substantial proportion (>10%) of the total chi-squared value (Supplementary Fig. 14E and Supplementary Table 5).^58^ Dark cells were the only category that met both these criteria. Therefore, we found that the IBA1 immunogold labelling was specific for the cell type we morphologically identified as dark cells, but not the aSyn immuno-positive dark cells, oligodendrocytes or neurons.

Given that the aSyn immuno-positive dark cells did not show specificity to IBA1 by immunogold labelling, we next attempted CLEM against multiple cell type-specific markers to investigate the possibility that these pathological cells could be a different type of oligodendrocyte, astrocyte or neuron. Free-floating sections were immunolabelled with commonly used antibodies for each cell type and imaged using fluorescent microscopy before being resin-embedded and CLEM sectioned for EM imaging. Given the challenges associated with reduced antigenicity in glutaraldehyde fixed tissue, we tested over 30 different antibodies and successfully found at least one antibody specific to each cell type that showed positive fluorescence staining (Supplementary Table 2). For each cell-specific marker, we could correlate the fluorescent signal for that cell type with an EM ultrastructure, however none of the cell type-specific markers used showed any immuno-reactivity to either the aSyn negative dark cells, or the aSyn positive dark cells (Supplementary Figs 15 and 16).

Owing to the difficulty of correlating large areas of fluorescence with the small fields of view obtained by TEM, an intermediate light microscopy step was included where ultrathin sections collected adjacent to the EM grids were stained with toluidine blue. The toluidine blue stained slides offered a larger field of view comparable to the areas imaged by fluorescence, with morphological details comparable to that obtained by TEM at low magnifications. This allowed the observation that the nuclei and cytoplasm of dark cells were clearly distinguishable by staining pattern and morphology from that of all other cell types whose identities were confirmed by CLEM using cell type-specific markers (Fig. 4). For the dark cells the toluidine blue stained both the heterochromatin and nucleoplasm, resulting in a dense and homogeneously stained nucleus. This phenotype was distinct from all other cell nuclei in the tissue where only the heterochromatin was stained, leaving a clear nucleoplasm. The nuclei of aSyn positive and aSyn negative dark cells were not distinguishable from each other by their toluidine blue staining pattern. Only after immunohistochemistry staining on adjacent sections could the aSyn positive dark cells be identified.

## Discussion

### GCIs in multiple system atrophy human brain contain fibrils, lysosomes and peroxisomes

In this study we describe three distinct types of aSyn immuno-positive inclusions found within oligodendrocytes, neurons, and dark cells from human post-mortem multiple system atrophy brains (Fig. 5). Consistent with previous studies,^6,7,60-66^ we show that GCIs are predominantly fibrillar; however, the GCI fibrils are intermixed with lysosomes and peroxisomes. While the presence of spherical profiles^2^ and dense bodies^60,63^ entrapped amongst the fibrils has been previously described, no significance had been attributed to them. A more recent study also described vesicles consistently co-localizing with aSyn pathology in multiple system atrophy brain, however the identity of these vesicles was not known.^78^ By confocal and electron microscopy, we confirmed the identity of these vesicles to be lysosomes and peroxisomes. Our observation of these organelles in over 100 GCIs studied by EM, supports the involvement of the autophagy-lysosomal pathway in multiple system atrophy.^79^

As autophagy has been shown to be essential for the differentiation, survival and myelination of oligodendrocytes,^80^ the presence of autophagy-related organelles in aSyn inclusions could be by-products of normal oligodendrocyte function. However, there is mounting evidence from genetic, *in vitro* and post-mortem brain studies suggesting that the autophagy-lysosomal system plays a crucial role in the degradation of aSyn,^81-85^ and that the disruption of such systems induces inclusion formation^83,86,87^ and human disease.^88-90^ While almost all GCIs contained lysosomes as well as multi-vesicular bodies, two inclusions from the putamen in particular appeared to have clusters of autophagy-related organelles enclosed by fibrils (Supplementary Fig. 1B, left and right). As the putamen is severely affected by degeneration in multiple system atrophy-Parkinson’s variant cases, further exploring the role of these organelles in GCI formation and investigating whether the number of autophagy-organelle clusters correlates with disease severity or more severely degenerated brain regions may aid in understanding the progression of inclusion formation.

The presence and clustering of peroxisomes in GCIs is a novel observation in our study, not previously described for multiple system atrophy. Peroxisomes can be degraded through the autophagy pathway^91-94^; therefore, their presence alongside other autophagy organelles could further support a role for the activation or perturbation of the autophagy pathway as a disease mechanism in multiple system atrophy. Peroxisomes are primarily involved in lipid- and reactive oxygen species metabolism, which in turn leads to a close association with mitochondria.^95^ Both organelles show altered age-related functions and have been linked to neurodegenerative disorders amongst others, such as Parkinson’s disease.^96-98^ However, despite genetic mutations implicating mitochondrial dysfunction in multiple system atrophy (reviewed by Compagnoni and Di Fonzo^99^), we did not observe any abnormal, disrupted or accumulated mitochondria in any of the GCIs. This suggests that mitochondrial dysfunction may not be linked to the formation of GCIs but may influence disease pathogenesis through a different mechanism.

Peroxisomes are also involved in the biosynthesis of myelin phospholipids,^100^ therefore their presence in aSyn GCIs could be a consequence of normal oligodendrocyte function. In contrast, their accumulation within GCIs could also be a sign of perturbed function, leading to the alterations in myelination commonly seen in multiple system atrophy patients.^101-103^ Their role in inclusion formation should thus be further explored in the future.

### NCIs in multiple system atrophy mimic neuronal pathology in Parkinson’s disease

Previous EM studies described perinuclear and globular NCIs to be indistinguishable from fibrillar GCIs.^2,6,21,62,63,65,104,105^ In contrast to those observations, we found NCIs to contain various ultrastructures that were clearly distinguishable from GCIs. We attribute this difference to the excellent tissue preservation we achieved using EM processing protocols that minimize ultrastructural loss commonly caused by sub-optimal tissue processing protocols,^106^ in combination with the short post-mortem delay at autopsy.

Two of the localized NCIs consisted of densely packed membranes, vesicles, and cellular organelles, where no fibrillar material could be detected. This inclusion was similar to the membranous inclusions we recently described for Parkinson’s disease tissue.^46^ The membranous NCI ultrastructure supports a previous suggestion of non-fibrillar ultrastructures for neuronal inclusions in multiple system atrophy based on different staining profiles across brain regions using silver staining compared to immuno-staining.^23^ However, we cannot rule out the possibility that some fibrillar material was visually obscured by the high density of vesicular packing within the inclusion.

The other NCIs consisted of a mixture of fibrillar and membranous material, and of note some showed an ultrastructure showing clustered mitochondria intermixed within the fibrils, in an arrangement similar to that which has been attributed to a pale body in Parkinson’s disease. Taken together, our observations of multiple ultrastructures for neuronal inclusions in multiple system atrophy suggest that it mimics the ultrastructural heterogeneity previously observed by EM for neuronal inclusions in the brain-stem of Parkinson’s disease donors.^46^ Our observations additionally support a previous immunohistochemistry study showing a pleomorphic spectrum of neuronal inclusions within multiple system atrophy patients.^22^ Despite observing ultrastructural similarities between neuronal inclusions in the multiple system atrophy donors compared to Parkinson’s disease, we did not observe any Lewy bodies that resembled the classic morphology of an electron dense core surrounded by a halo of radiating fibrils. As far as we know, there has been no EM data of these structures from multiple system atrophy brain reported in the literature to date; however, their presence in multiple system atrophy brain has been intimated by their histological or immunological staining profile in paraffin sections. Given that Lewy bodies are not reported to be a prominent feature of MSA pathology, it is not surprising that we did not observe these structures given the additional scarcity of neuronal inclusions in multiple system atrophy brain.

The fact that the ultrastructure of NCIs were clearly distinguishable from GCI pathology supports a hypothesis for a different assembly mechanism for aSyn accumulation in neurons compared to oligodendrocytes.^21^ Further ultrastructural studies of other neuronal accumulations by CLEM are therefore needed to broaden our understanding of this spectrum of inclusions, and the contribution of membranes and fibrils to this pathology. Our finding of morphologically similar neuronal pathologies in multiple system atrophy and Parkinson’s disease provides an ultrastructural link between the two diseases, which needs further exploration.

### Dark cells are likely to be dark microglia

Finally, we describe the presence of aSyn inclusions in cells which had a distinctive condensed and misshapen nucleus and highly electron dense nucleo- and cytoplasm. To our knowledge, aSyn pathology in such ‘dark’ cells have not previously been described for multiple system atrophy, or any other synucleinopathy. Although we present 47 aSyn immuno-positive dark cells in this study, we observed many more of these cells (both aSyn immuno-positive and immuno-negative) occurring frequently in the tissue. Given their abundance within the brain donors we studied, we found it surprising that this pathology had not been previously described.

Similar cells matching this phenotype have been recently characterized as ‘dark microglia’ and have been associated with the cell being in a pathological state.^74-77^ As information on dark microglia in human brain is scarce, we sought to confirm that the novel aSyn immuno-positive cells we identified in our study were also of microglial origin. By immunogold labelling, we found that the aSyn immuno-negative dark cells showed preferential labelling for the common microglial marker IBA1, while the aSyn immuno-positive dark cells did not. Furthermore, our CLEM experiments using additional microglial antibodies and markers for different cell types also failed to conclusively confirm the cellular identity of the suspected dark microglia.

Our results are supported by previous publications reporting that the common microglial proteins are down-regulated in dark microglia, making them difficult to detect by antibody-based microscopy methods.^77,107^ It is also possible that the antigenicity of the dark cells is more affected by the presence of glutaraldehyde in the tissue fixative than for other cell types, or that they are immuno-positive for other antibodies that were not able to be used in this study (such as the microglial markers CD11b, CD45 and TREM2 for which we could not find a working antibody). Finally, from our immuno-histochemistry labelling on ultra-thin sections obtained by CLEM, we observed that the whole microglial cell body was immuno-positive for aSyn. Therefore, it is possible that the high-level of aSyn in these cells is disrupting the protein homeostasis of IBA1 in some way, so that it is less likely to be detected in these cells using immuno-labelling. However, since no protein marker is currently available to unambiguously identify dark cells as dark microglia, their distinctive phenotype remains the only method available to differentiate them from other cell types.

These differences between the dark cells and other cell types were emphasized in our study through a comparison of their morphologies using toluidine blue staining. The condensed nucleo- and cytoplasm of the dark cells were clearly distinguishable from that of the other cell types, whose identities were confirmed by immuno-fluorescence-based CLEM. However, in these images, it was not possible to differentiate between the aSyn immuno-positive dark cells and aSyn immuno-negative dark cells.

The aSyn immuno-positive dark cells were identified by immunohistochemistry using antibodies that detect regions within amino acids 15–123 of aSyn (clone 42, BD Biosciences; LB509, Life Technologies). However, it is important to note that pathological studies using markers against specific epitopes of aSyn not included in this region, such as pS129, N-terminal or C-terminal antibodies, may result in a different staining pattern of pathology within the tissue.^108^ Collectively, these findings may explain why aSyn immuno-positive dark cells have not previously been reported in the literature and underscore the need to identify a marker capable of specifically identifying these dark cells and distinguishing them from other cell types. While we cannot definitively exclude the possibility that these pathological cells belong to another cell type, all available morphological evidence supports their classification as dark microglia.

### Dark cells contain GCI-like fibrils and could be important for disease progression

We show here that the ultrastructure of dark cells can be GCI-like, where they contain 22 nm fibrils intermixed with autophagy organelles, or non-GCI-like, where a specific ultrastructure that could account for the aSyn immuno-positive staining was not recognizable. Our finding of GCI-like fibrillar dark cells could indicate the uptake of extracellular aSyn fibrils as has been observed in the case of astrocytes in human brain.^82^ However, our observation of mature fibrils appearing to spread between an oligodendrocyte and a dark cells suggests that there could also be direct cell-to-cell transfer of aSyn fibrils/fibril seeds between microglia and GCIs. We did not observe any boundary membrane between the two cells, and there was also no membrane enclosing the fibrils. Rather, the fibrils inside the space of the dark cells were continuous with the GCI in the oligodendrocyte. This suggests that one possible mechanism for the spread of disease pathology between cell types may be through cytoplasmic fusion.

In contrast to the fibrillar dark cells, in other dark cells no recognizable ultrastructure accounted for the presence of accumulated aSyn in those cells. As accumulated pathological aSyn fibrils are easily observed by TEM, it is possible that aSyn is present in these dark cells in a non-fibrillar form. As such, the presence of accumulated non-fibrillar aSyn in dark cells could be evidence for: (i) the spontaneous accumulation of endogenous aSyn in dark cells representing a precursor to the formation of GCI-like fibrillar dark cells; (ii) the accumulation of transmitted and toxic aSyn seeds, and a precursor to the formation of GCI-like fibrillar dark cells; or (iii) microglial degradation of engulfed aSyn fibrils from GCIs resulting in accumulated non-fibrillar aSyn. Nevertheless, our observation of various types of ultrastructures for dark cells suggests that we captured different stages of aSyn accumulation, or that different mechanisms of aSyn accumulation could be occurring in these cells.

Interestingly, we have only identified pathological dark cells in multiple system atrophy cases and not in controls or other synucleinopathies when performing CLEM using the same antibodies for aSyn (unpublished data). However, this observation warrants comprehensive evaluation in future studies, particularly when an identifying dark cells marker becomes available. Microglial uptake of endogenous aSyn has been suggested as a mechanism of disease transmission,^109-111^ and since microgliosis has been shown to be a major disease phenomenon in multiple system atrophy,^112-116^ further investigation of its role in disease may be important for understanding the disease mechanism and progression.

## Summary

Our results highlight the ultrastructural diversity of aSyn pathology in multiple system atrophy brain. We found that fibrillar GCIs consistently co-localize with the autophagy organelles lysosomes and peroxisomes. Neuronal inclusions mimic the ultrastructural heterogeneity previously identified for brain-stem neuronal inclusions in Parkinson’s disease in that they were completely membranous or contained a mixture of fibrillar and membranous material. The observation of fibrillar and non-fibrillar ultrastructures in aSyn immuno-positive dark cells, suggests a role for these cells in disease pathogenesis. Further studies are now required to comprehensively characterize cell type and cell-stage specific aSyn accumulation in multiple system atrophy brain, which will have important implications for the understanding of disease pathogenesis and aSyn aggregation.

## Supplementary Material

awae137_Supplementary_Data

## Data Availability

All images will be made available on request.
