# Supplementary material for: Distinct ultrastructural phenotypes of glial and neuronal alpha-synuclein inclusions in multiple system atrophy
Source: Brain. 2024 May 2;147(11):3727–41. doi: 10.1093/brain/awae137 (PMC11531854; doi:10.1093/brain/awae137)
Supplement: awae137_Supplementary_Data [file awae137_supplementary_data.zip › brain-2023-00776-File007.pdf]

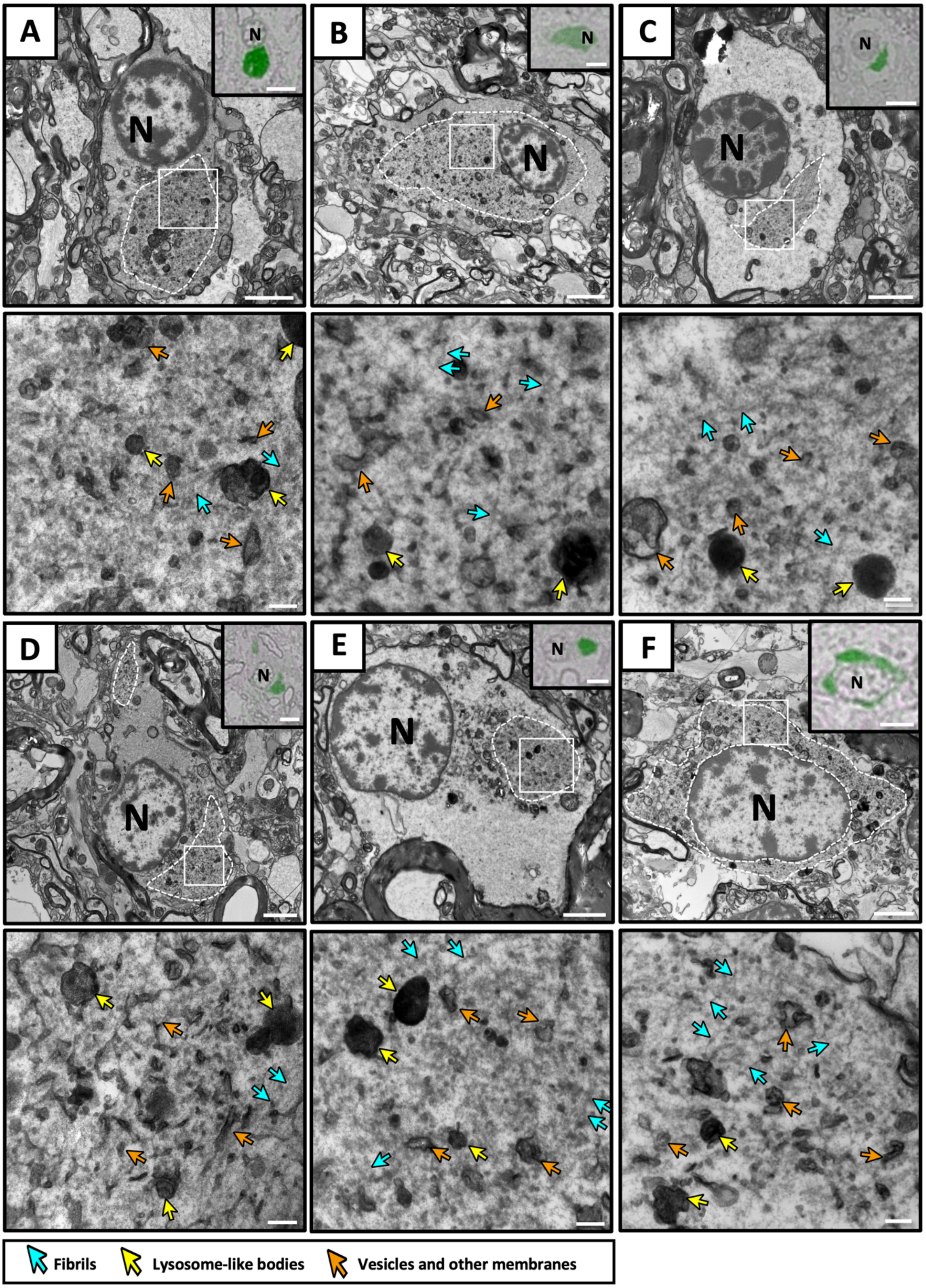

Supplementary Figure 1 Further examples of GCIs localized by CLEM. TEM micrographs of each inclusion is shown at low and high magnification. Light microscopy images of aSyn immuno-staining (green) used to identify GCIs (white dotted line) are shown in the inset. Inclusions shown in **A-E** are from the SN of Donor D, **F** is from the SN of Donor G. N = nucleus. Scale bars: EM low mag 2 µm, high mag 200 nm; LM 2 µm.

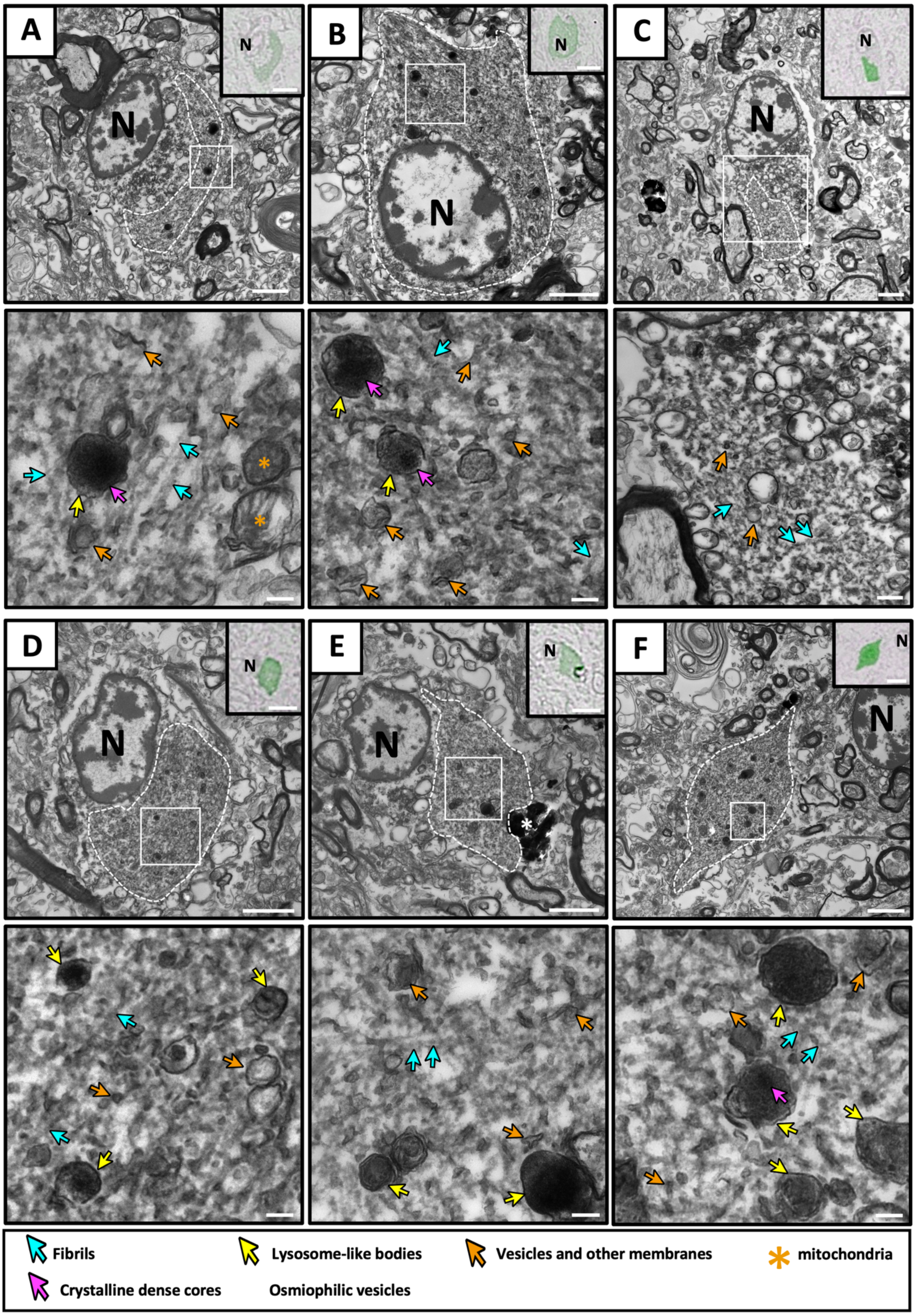

Supplementary Figure 2 Further examples of GCIs from the SN of Donor C localized by CLEM. TEM micrographs of each inclusion is shown at low and high magnification.Light microscopy images of aSyn immuno-staining (green) used to identify GCIs (white dotted line) are shown in the inset. N = nucleus. Scale bars: EM low mag 2 µm, high mag 200 nm; LM 2 µm.

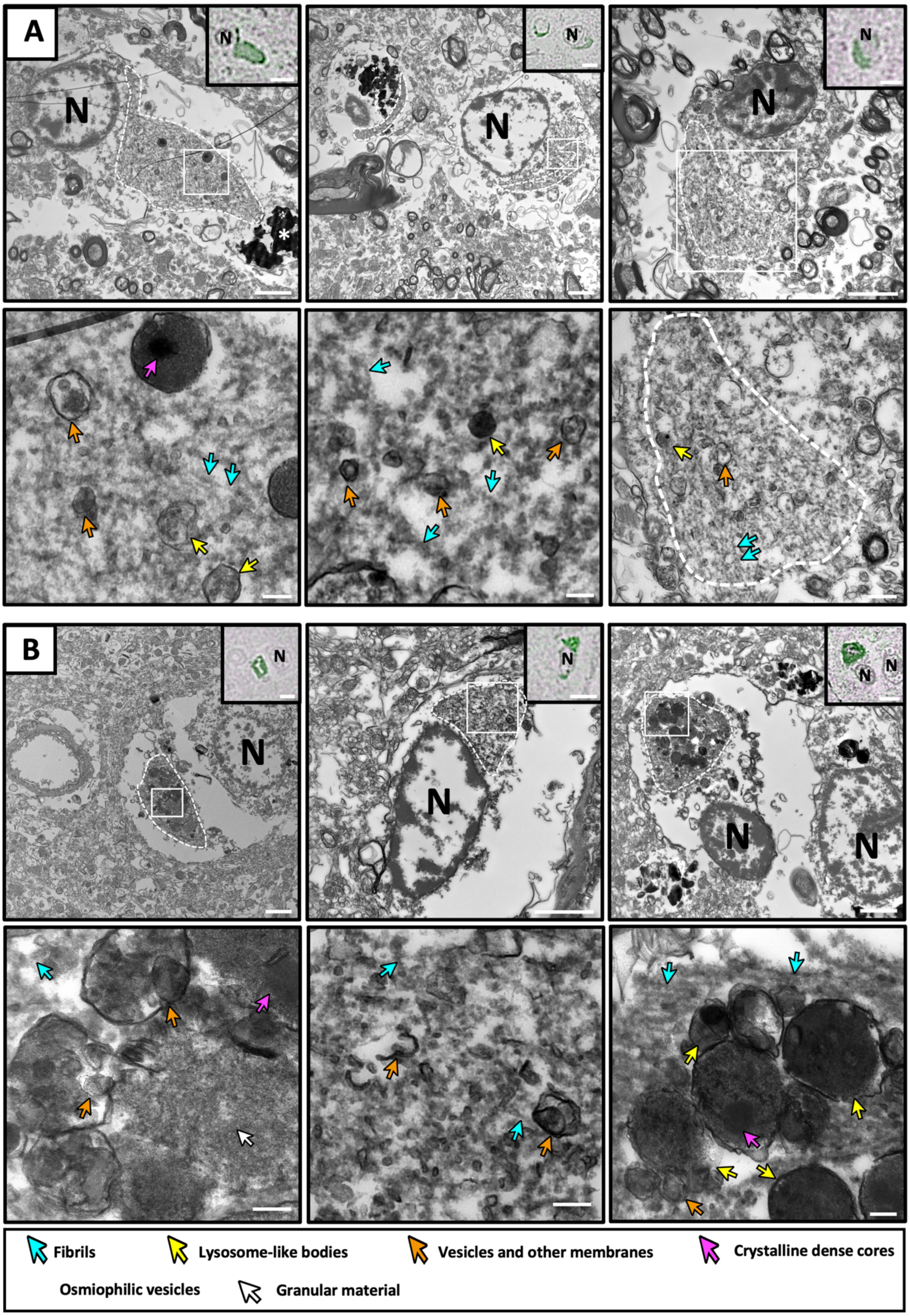

Supplementary Figure 3 Further examples of GCIs from the PUT localized by CLEM. TEM micrographs of each inclusion is shown at low and high magnification.Light microscopy images of aSyn immuno-staining (green) used to identify GCIs (white dotted line) from Donor B **(A)** and Donor C (**B)** are shown in the inset. N = nucleus. Scale bars: EM low mag 2 µm, high mag 200 nm; LM 2 µm.

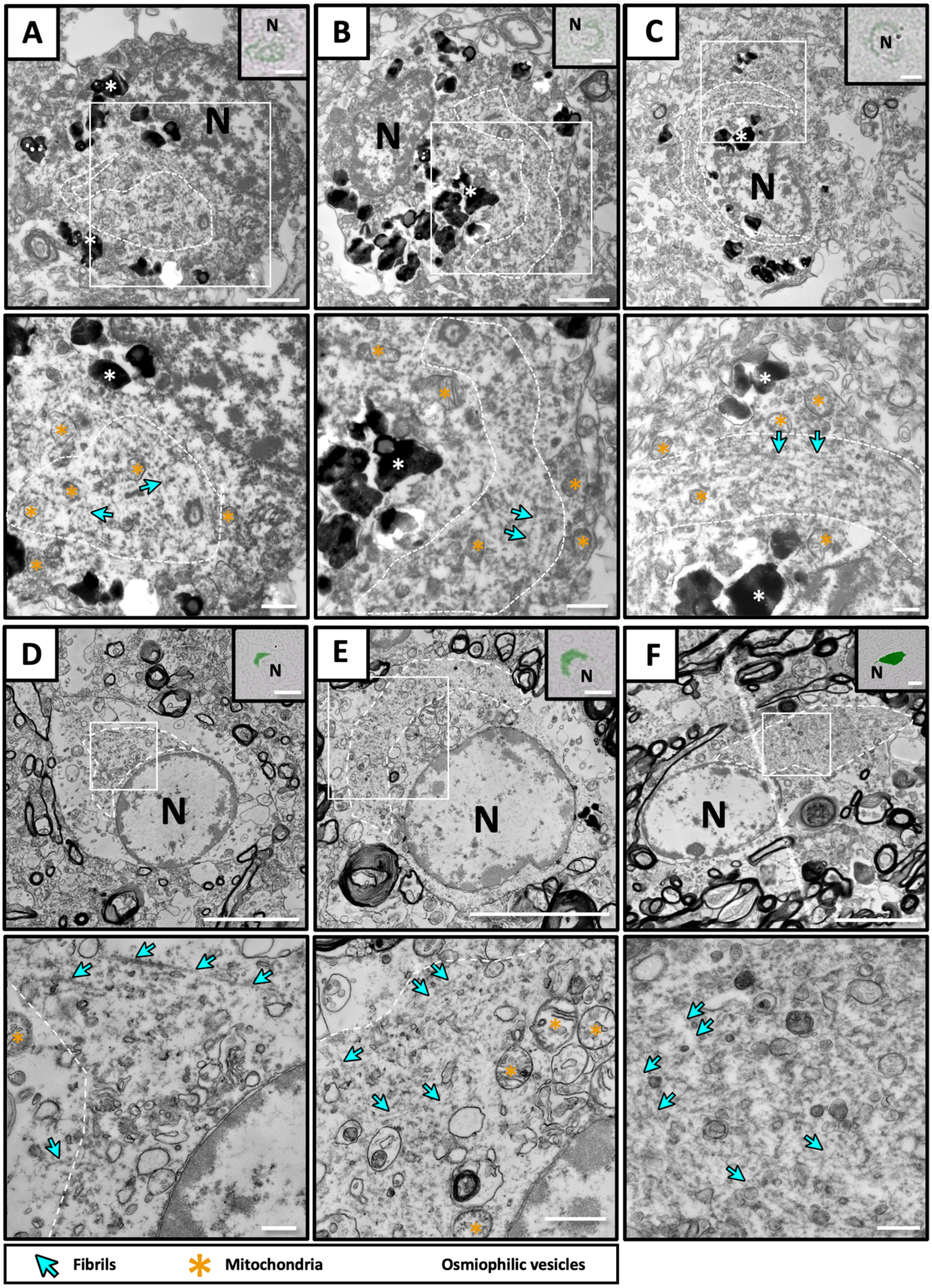

Supplementary Figure 4 Further examples of GCIs from the PUT localized by CLEM. TEM micrographs of each inclusion is shown at low and high magnification.Light microscopy images of aSyn immuno-staining (green) used to identify GCIs (white dotted line) from Donor A **(A-C)** and Donor G **(D-F)** are shown in the inset. N = nucleus. Scale bars: EM low mag 2 µm, high mag 500 nm; LM 2 µm.

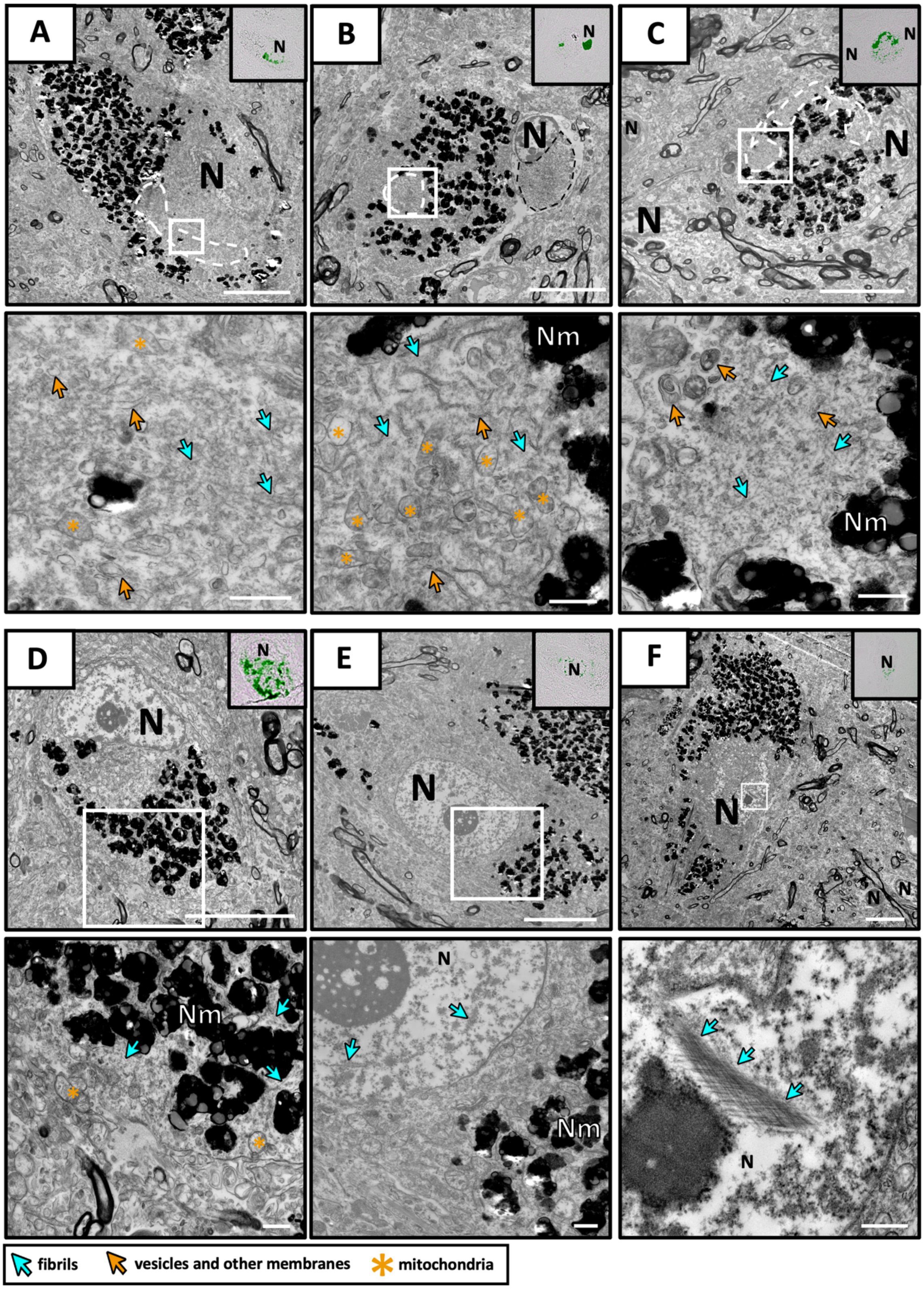

Supplementary Figure 5 Further examples of NCIs from the SN localized by CLEM from Donor F. TEM micrographs of each inclusion is shown at low and high magnification. The neuron in **(B)** is adjacent to an oglio containing a GCI (black dotted line). The inclusion shown in **(D)** show fibrils intermixed amongst the neuromelanin. The neuron in **(E)** also contained nuclear fibrils. The neuron in **(F)** showed only an NNI. Light microscopy images of aSyn immuno-staining (green) used to identify NCIs (white dotted line) and NNIs are shown in the inset. N = nucleus. Nm = neuromelanin Scale bars: EM low mag 10 μm, high mag 1 μm.

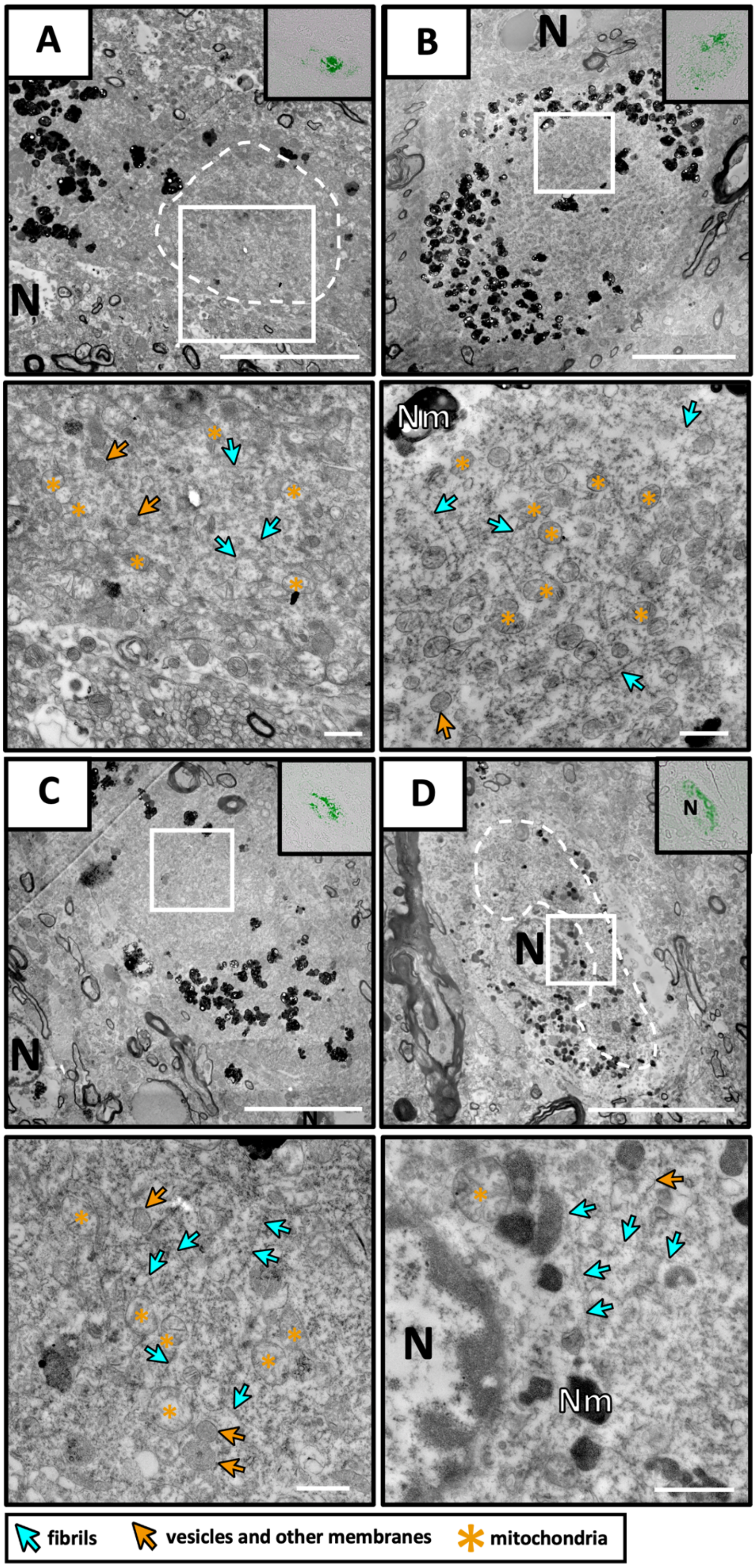

Supplementary Figure 6 Further examples of NCIs from the SN localized by CLEM from Donor H. TEM micrographs of each inclusion is shown at low and high magnification. Light microscopy images of aSyn immuno-staining (green) used to identify NCIs (white dotted line) are shown in the inset. N = nucleus. Scale bars: EM low mag 10 µm, high mag 1 µm.

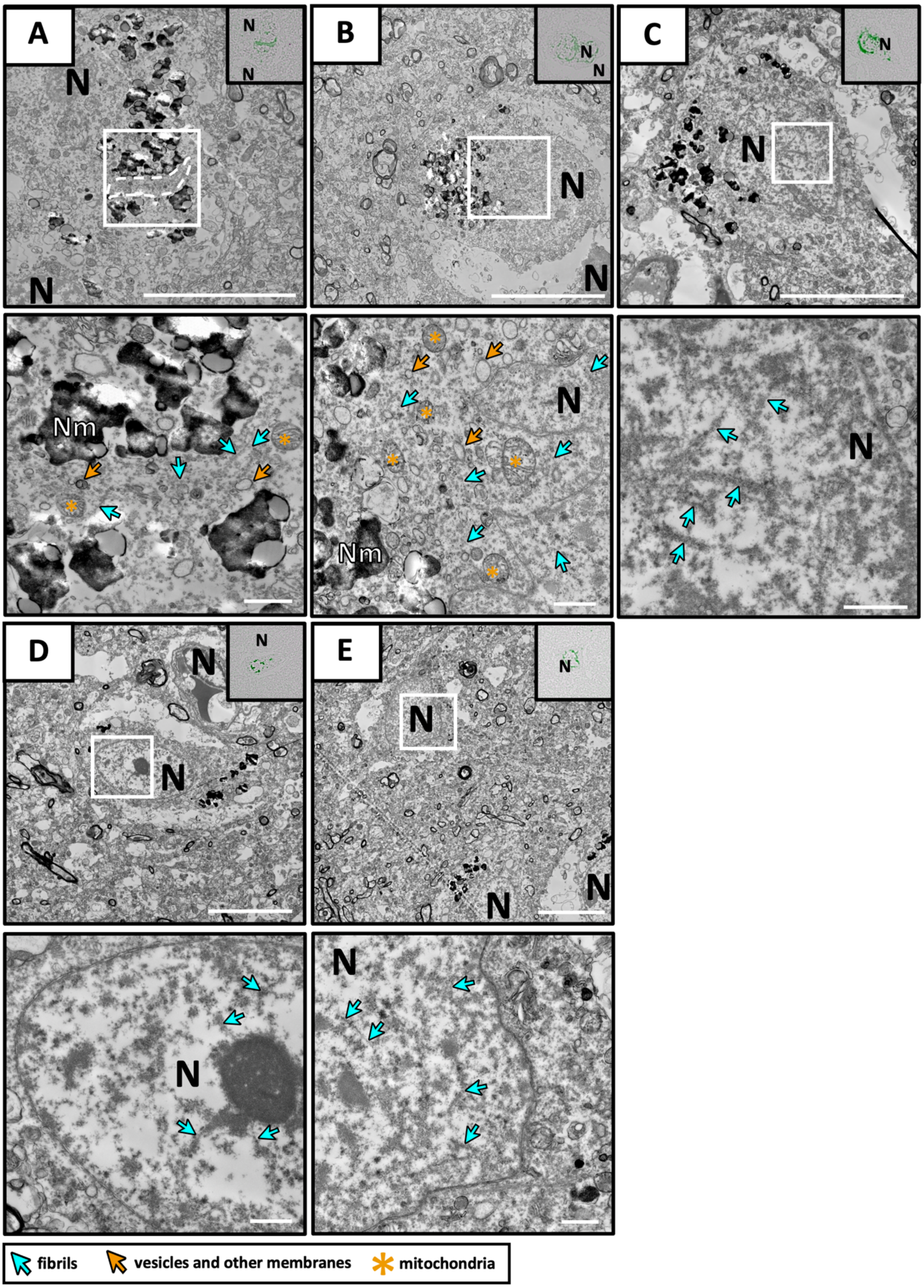

Supplementary Figure 7 Further examples of NCIs from the PUT localized by CLEM from Donor G. TEM micrographs of each inclusion is shown at low and high magnification. The neuron in **(B)** also contained nuclear fibrils. In neurons (**C-E**) only NNIs were evident. Light microscopy images of aSyn immuno-staining (green) used to identify NCIs (white dotted line) and NNIs are shown in the inset. N = nucleus. Nm = neuromelanin. Scale bars: EM low mag 10 µm, high mag 1 µm.

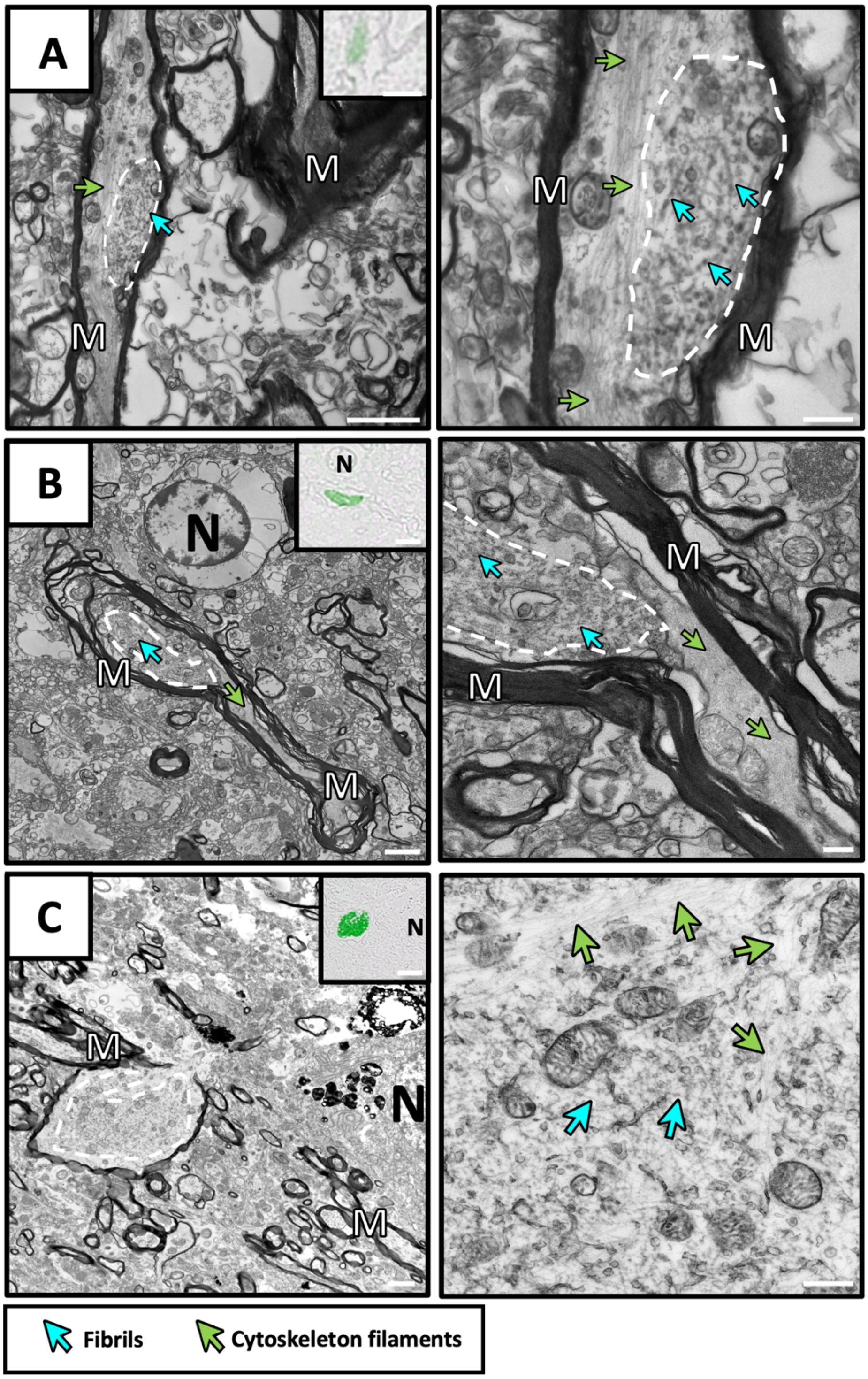

Supplementary Figure 8 aSyn immuno-positive inclusions inside an axon visually underlines the structural difference between cytoskeleton filaments and aSyn immuno-positive fibrils. TEM micrographs of aSyn immuno-positive axons localized by CLEM from the SN of Donor C **(A)**, Donor D **(B)** and Donor F **(C)**. The aSyn fibrils (blue arrows) within the immuno-positive region (white dotted line) can be seen crisscrossing one another and are distinct from the cytoskeletal filaments (green arrows) which appear to run parallel throughout the axon and are visibly thinner. Light microscopy image showing aSyn immuno-positive staining (green) shown in inset. M = myelin. Scale bars: EM 2 µm (left), 0.5 µm (right)**;** LM 7 µm.

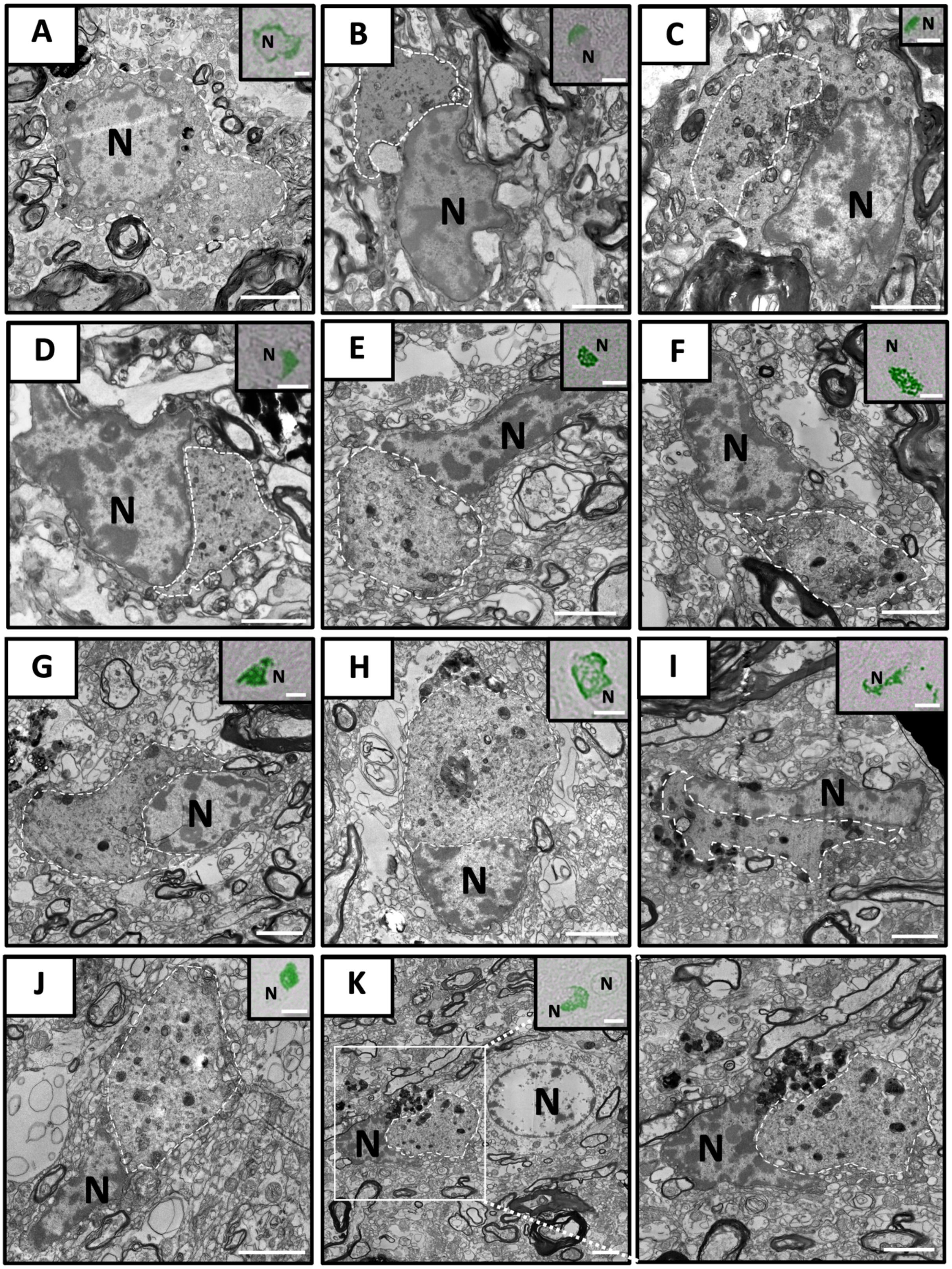

Supplementary Figure 9 Further examples of dark cells localized by CLEM. TEM micrographs of each inclusion are shown. LM images of aSyn immuno-staining (green) used to identify dark cells from the SN of Donor G **(A)** and Donor D **(B-K)** are shown in the inset. **(K)** shows an aSyn immuno-positive dark cell adjacent to an oligodendrocyte. N = nucleus. Scale bars: 2 µm.

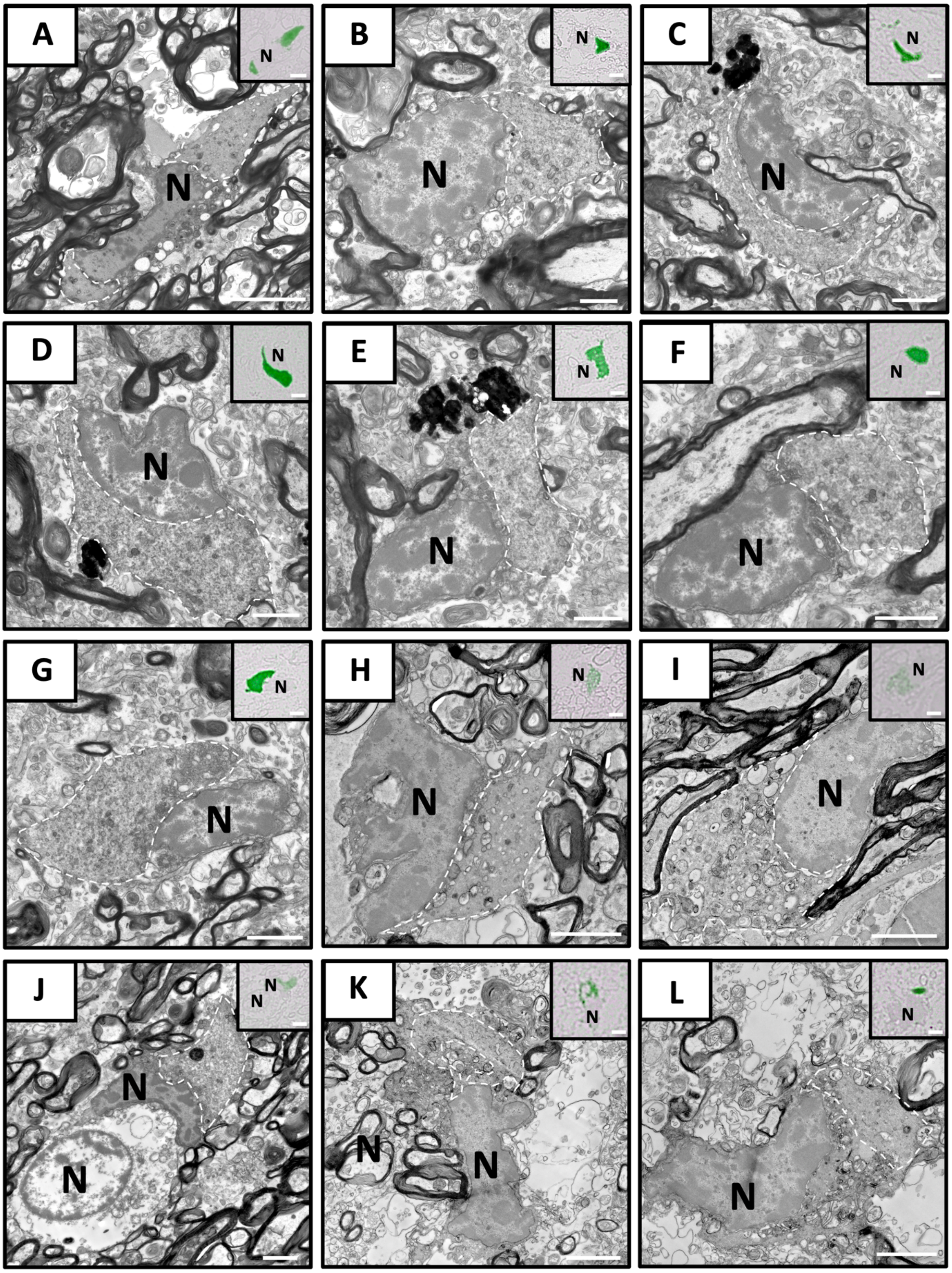

Supplementary Figure 10 Further examples of dark cells localized by CLEM. TEM micrographs in the SN of Donor F **(A-I)** and PUT of Donor G **(J-L)**. **(J)** shows an aSyn immuno-positive dark cell adjacent to an oligodendrocyte. LM images of aSyn immuno-staining (green) used to identify dark cells are shown in the inset. Scale bars 2 µm.

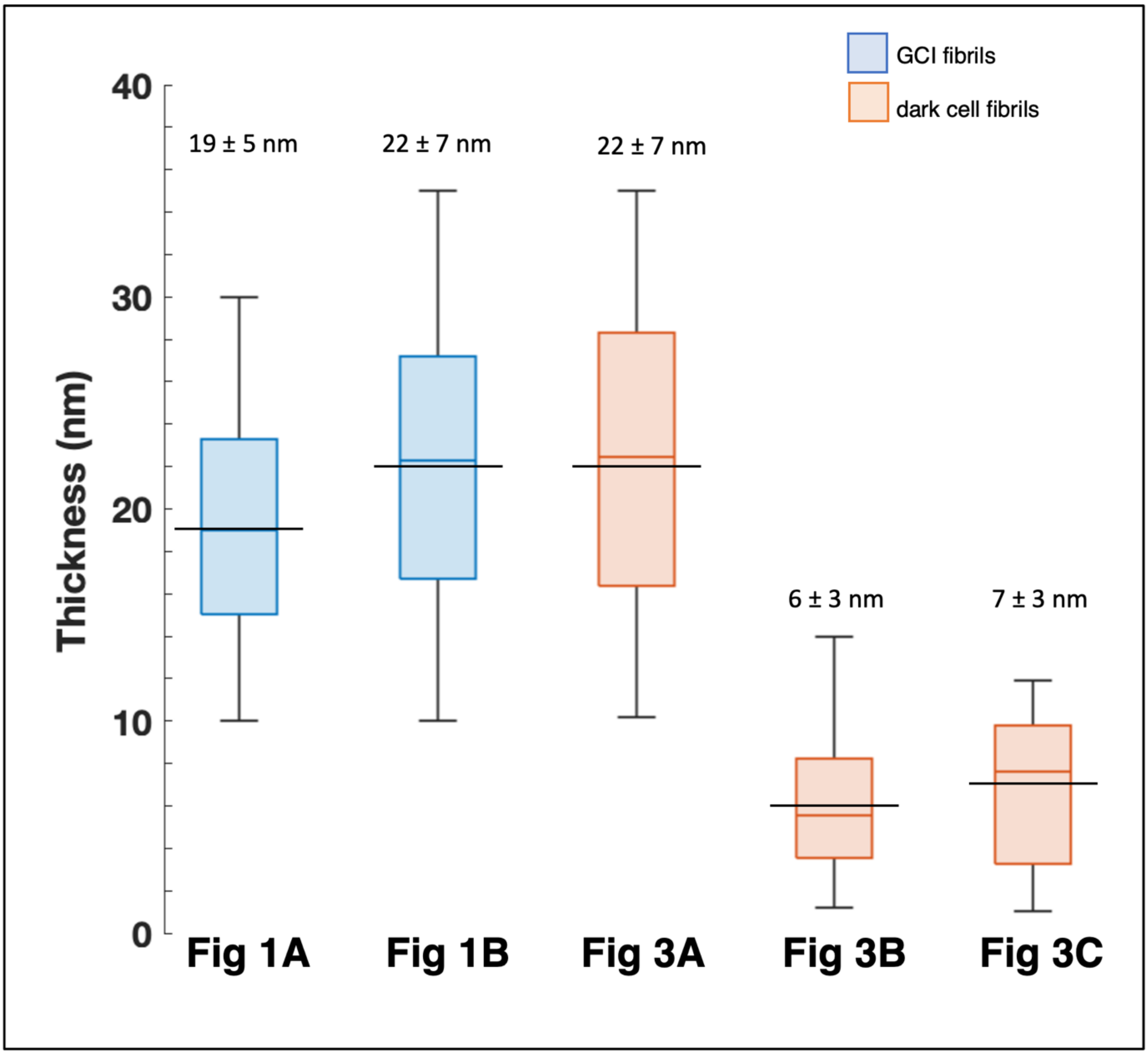

Supplementary Figure 11 Box plots showing the distribution of fibril thickness (width) extracted from the segmented tomograms. For each tomogram, the median, range and average (black line) is shown. The figure number relating to each tomogram as well as the average fibril width +/- standard deviation is indicated.

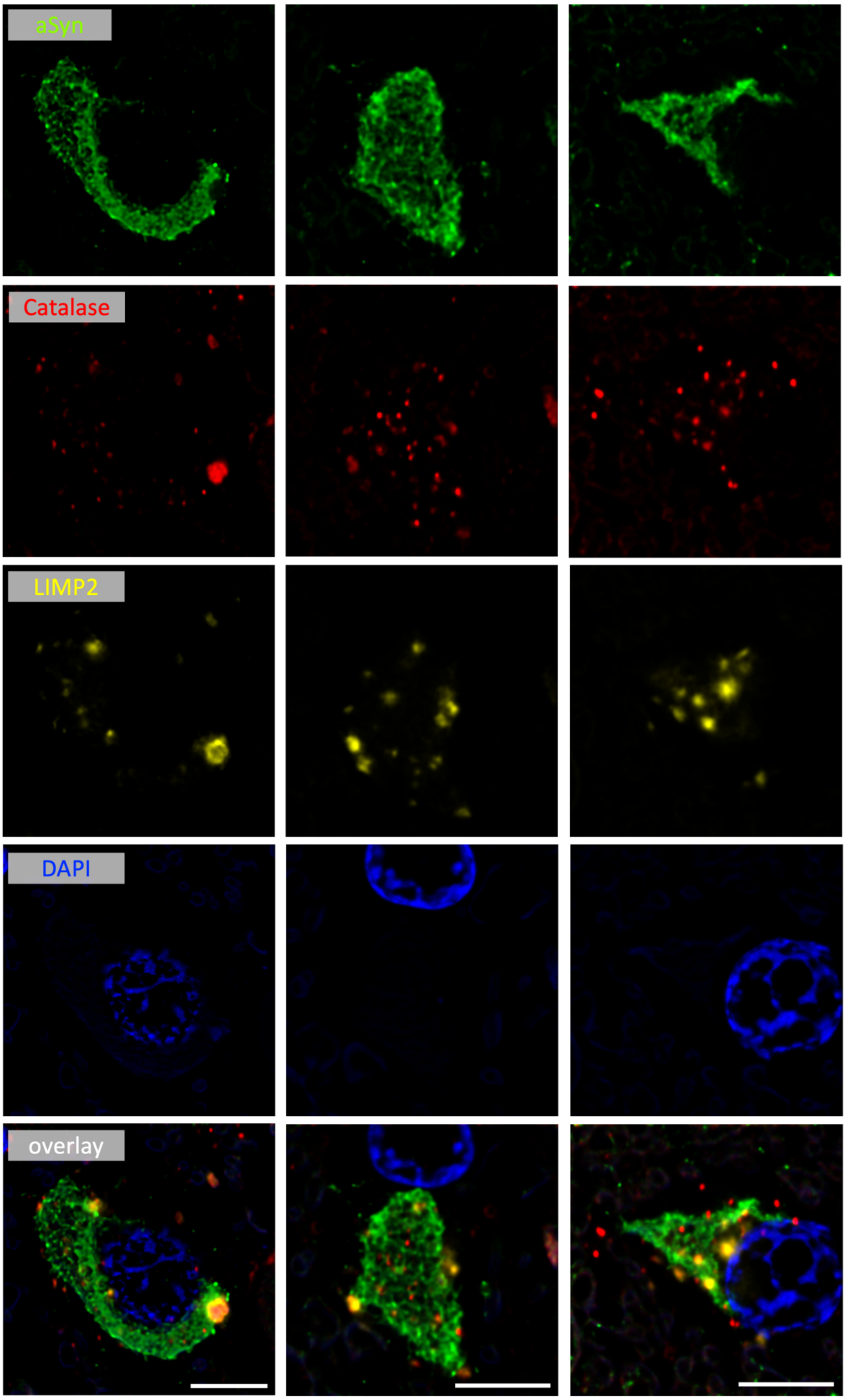

Supplementary Figure 12 Lysosomes and peroxisomes co-localize with GCIs. Confocal microscopy of GCIs (aSyn; green) localized within Donor E were immunolabelled to show peroxisomes (catalase; red), lysosomes (limp2; yellow) and cell nuclei (DAPI; blue). The overlay shows the co-localization of each marker within the GCI. Scale bars: 5 µm.

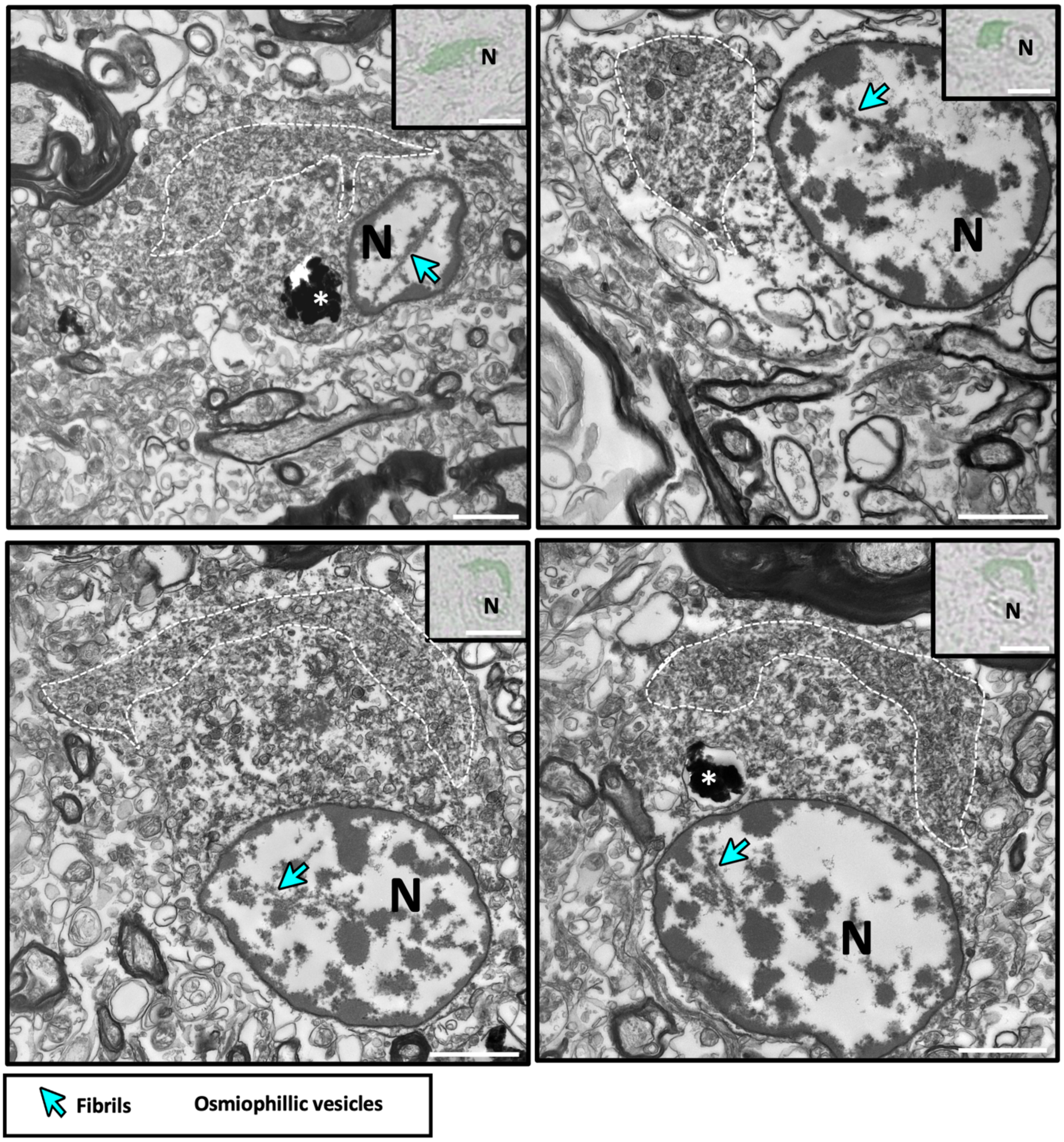

Supplementary Figure 13 Nuclear fibrils in GCI containing cells. The inclusions were localized from the SN in Donor C. Light microscopy images of aSyn immuno-staining (green) used to identify GCIs (white dotted line) are shown in the inset. N = nucleus. Scale bars: EM 2 µm; LM 5 µm.

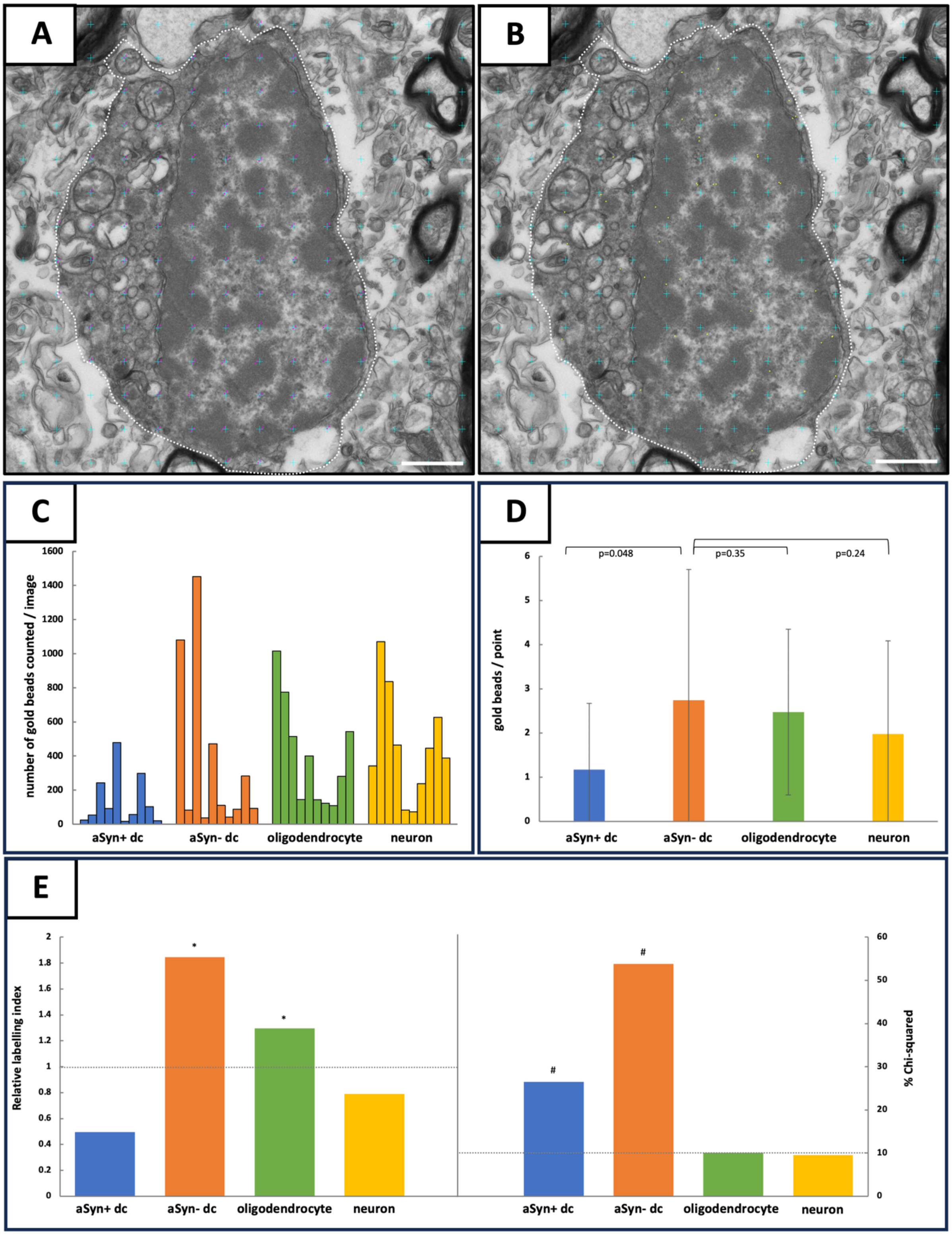

Supplementary Figure 14 Quantification of immunogold labelling against IBA1. TEM micrograph of a dark cell (dc; white dotted outline) that has been immuno-labelled with IBA1 and detected with 10 nm gold beads. **(A)** A grid (aqua crosshairs) was superimposed over the image and the crosshairs (points) falling within the boundary of the cell were counted (magenta dots) to determine the area of the cell. **(B)** The gold beads detected within the same boundary (yellow dots). **(C)** The total number of gold beads counted for each of the 10 images taken per cell-type shows the large variability of the number of gold beads detected for each

image within each cell-type. **(D)** The average number of gold beads/point +/- the standard deviation shows dark cells to have the highest labelling density for IBA1. A student one-tailed t-test comparing the IBA1 labelling density of dark cells to oligodendrocytes and neurons revealed the differences to be insignificant ($p > 0.05$), but the difference between immuno-positive (aSyn+) and immuno-negative dark cells (aSyn-) to be significant ($p < 0.05$). **(e)** The preferential labelling of IBA1 was determined using the relative labelling (left), and the partial Chi-squared value (right). Labelling was determined to be specific on the basis of satisfying two criteria: A relative labelling index >1 and, a corresponding partial Chi-squared value accounting for >10% (#) of the total Chi-squared. aSyn- dark cells were the only cell-type which satisfied both criteria. Scale bar: EM 1 µm

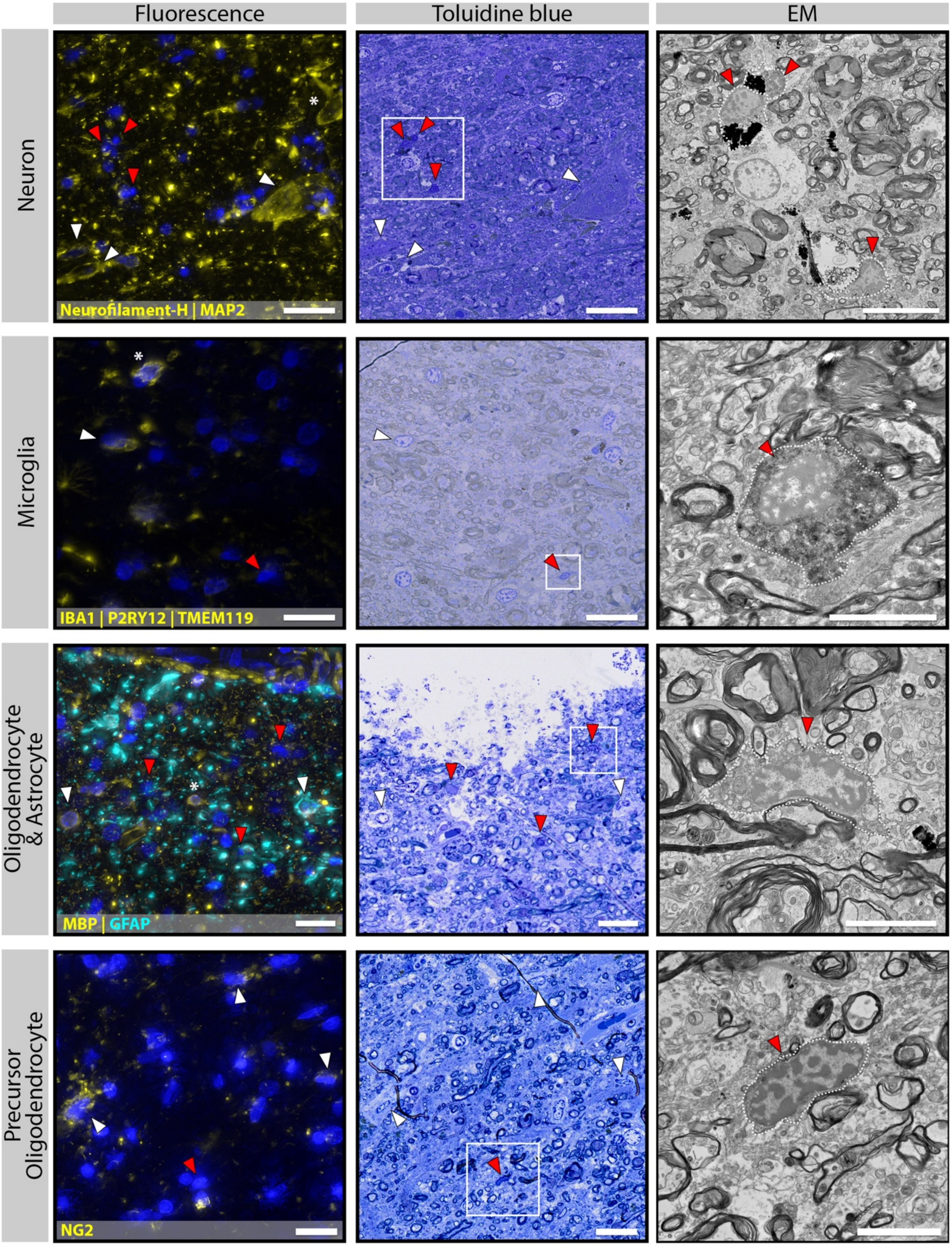

Supplementary Figure 15 dark cells are not immuno-positive for common cell-type specific markers. CLEM was performed in the SN of Donor G to identify cells immuno-positive for neuronal markers (neurofilament-H and MAP2 antibody cocktail), microglia (IBA1, P2RY12 and TMEM119 antibody cocktail), astrocytes (GFAP antibody), oligodendrocytes (MBP antibody), and precursor oligodendrocytes (NG2 antibody). For each cell type the maximum projection of a fluorescence z-stack taken within a free-floating 30 µm brain slice is shown. Following

resin embedding and CLEM sectioning, the same region has been correlated to 200 nm sections collected on glass slides and stained with toluidine blue, and the same cell was imaged by electron microscopy (EM) on a consecutive grid. Cells stained by indicated protein markers and correlated in the adjacent toluidine blue section are marked with white arrowheads. Cells stained by the indicated protein markers but located in a different z-plane above or below the shown adjacent toluidine blue section are denoted with white asterisks. Dark cells, indicated by red arrowheads showed no immuno-positive staining for any of the indicated antibodies. The white box signifies the area magnified for the toluidine blue staining. White dotted line traces the outline of the dark cells in the EM. Scale bars: fluorescence and toluidine blue 20 µm; EM neuron 10 µm; all other EM 4 µm.

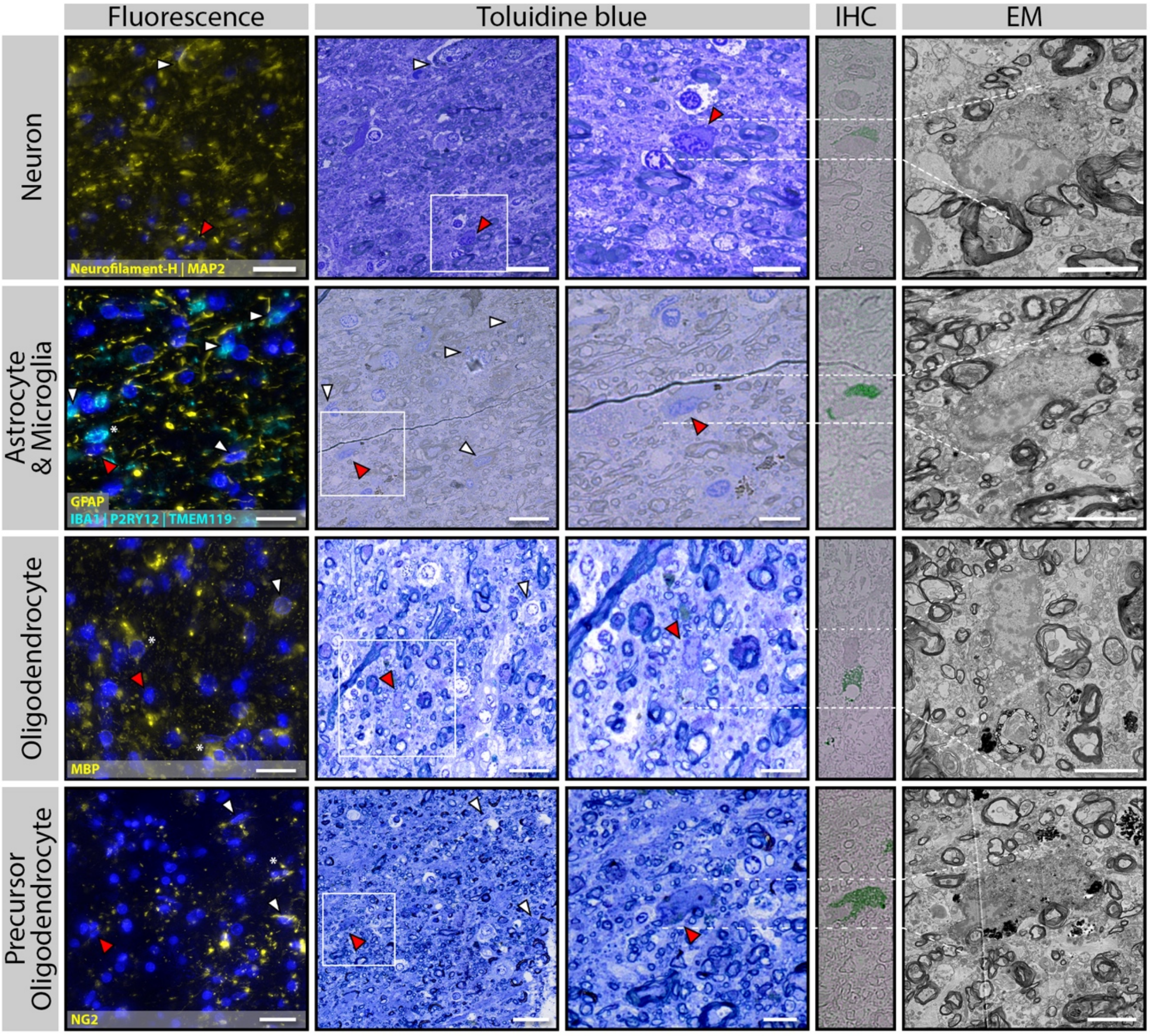

Supplementary Figure 16 dark cells are not immuno-positive for common cell-type specific markers. CLEM was performed in the SN of Donor G to identify cells immuno-positive for neuronal markers (neurofilament-H and MAP2 antibody cocktail), microglia (IBA1, P2RY12 and TMEM119 antibody cocktail), astrocytes (GFAP antibody), oligodendrocytes (MBP antibody), and precursor oligodendrocytes (NG2 antibody). For each cell type the maximum projection of a fluorescence z-stack taken within a free-floating 30 µm brain slice is shown. Following resin embedding and CLEM sectioning, the same region has been correlated to 200 nm sections collected on glass slides and stained with toluidine blue. aSyn immunohistochemistry

(IHC) was performed on adjacent sections to identify the immuno-positive dark cells (green), and the same cell was imaged by electron microscopy (EM) on a consecutive grid. Cells stained by indicated protein markers and correlated in the adjacent toluidine blue section are marked with white arrowheads. Cells stained by the indicated protein markers but located in a different z-plane above or below the shown adjacent toluidine blue section are denoted with white asterisks. Dark cells, indicated by red arrowheads showed no immuno-positive staining for any of the indicated antibodies. The white box signifies the area magnified for the toluidine blue staining. White dashed lines trace the dark cells through adjacent sections for the toluidine blue stain, the IHC, and the EM. Scale bars: fluorescence and low magnification toluidine blue: neuron, microglia & astrocyte, oligodendrocyte - 20 µm; precursor oligodendrocyte - 30 µm; high magnification toluidine blue and IHC 10 µm; EM 5 µm.
