# Supplementary material for: Distinct ultrastructural phenotypes of glial and neuronal alpha-synuclein inclusions in multiple system atrophy
Source: Brain. 2024 May 2;147(11):3727–41. doi: 10.1093/brain/awae137 (PMC11531854; doi:10.1093/brain/awae137)
Supplement: awae137_Supplementary_Data [file awae137_supplementary_data.zip › brain-2023-00776-File008.pdf]

Ultrastructure of multiple system atrophy brain pathology

Tables

Supplementary Table 1 Demographic details and neuropathological features of the included MSA-P brain donors.

Donor ID	Clinical diagnosis	Pathological diagnosis	age at death (y)	F/M	PMD (hrs ± min)	Brain weight (g)	ABC	Braak aSyn stage	Braak NFT stage	Thal Phase*	CAA	Other	Experiment donor was used for:
A	MSA-P	MSA-P	74	M	3:50	1350	A3B1C1	4	3	4	Type 1, stage 1/3	Slight ARTAG	CLEM
B	MSA-P	MSA-P	84	F	4:10	1175	A2B1C1	3	1	4	Type 2, stage 1/3	Severe ARTAG	CLEM
C	MSA	MSA-P	49	F	4:15	1120	A0B0C0	3	0	0	-	-	CLEM
D	MSA	MSA-P	84	M	5:40	1355	A1B1C0	3	1	0	-	-	CLEM, immunogold
E	MSA	MSA-P	66	M	4:55	1391	A0B1C0	0	1	0	-	-	Confocal microscopy
F	MSA-P	MSA-P	64	F	4:00	1185	A0B1C0	3	1	0	0	infarcts	CLEM, immunogold
G	MSA-P	MSA-P	68	M	4:45	1590	A1B1C0	6	1	1	Type 2, stage 2/3	-	CLEM
H	MSA-P	MSA-P	67	F	5:20	1200	A2B1C0	4	2	4	0	-	CLEM

y= year; F/M=female/male; PMD= postmortem delay; g=grams; NFT= neurofibrillary tangle. ABC score[38], Braak aSyn stage[3], Braak NFT stage[2, 93], Thal Phase[4], CAA[4]; ARTAG[49].

Supplementary Table 2 List of antibodies tested for fluorescence CLEM

Target	Antibody	Clone	Host Species	Company	Product number	Outcome	Dilution used
Microglia	IBA1		Rabbit	WAKO	019-19741	Specific staining	1/500
			Goat	Abcam	ab5076	Specific staining, weak	1/100
	CD11b	EPF13444	Rabbit	Abcam	ab133357	No staining	
		5C6	Rat	BioRad	MCA711G	No staining	
		238446	Mouse	Novus Biologicals	MAB16991	Stained astocytes, not microglia (100% overlap with GFAP staining)	
	CD45	IBL-3/16	Rat	BioRad	MCA1388	No staining	
		EP322Y	Rabbit	Abcam	ab40763	Stained astocytes, not microglia (100% overlap with GFAP staining)	
	P2RY12		Rabbit	Sigma/Merck	HPA014518	Specific staining	1/100
			Rabbit	Alomone labs	APR-012	No staining	
	CD68	KP1	Mouse	Invitrogen	14-0688-82	Non-specific staining	
		FA-11	Rat	Biorad	MCA1957	Staining of cellular processes only	1/100
			Rabbit	Cell Signalling Technologies	76437	Weak staining of cellular processes only	
	TMEM 119	28-3	Rabbit	Abcam	ab209064	No staining	
		C-terminal	Rabbit	Abcam	ab185333	Specific staining	1/100
		106-6	Rabbit	Abcam	ab2010405	No staining	
			Rabbit	Sigma/Merck	HPA051870	Weak staining	
	TREM2	2B5	Mouse	Novus Biologicals	NBP1-07101SS	no signal	
Oligodendrocytes	MBP		Chicken	Life Technologies	PA1-10008	Specific staining	1/100
			Rabbit	Abcam	ab218011	No staining	
	Olig-2		Rabbit	Sigma/Merck	ab9610	Staining of cellular processes only	1/100
			Goat	R&D Systems	AF2418	No staining	
			Rabbit	Abcam	ab109186	No staining	
	SOX10		Rabbit	Abcam	ab227680	No staining	
			Rabbit	Abcam	ab155279	No staining	
	MOG		Rabbit	Abcam	ab109746	No staining	
Pre-cursor oligodendrocytes	NG2		Rabbit	Abcam	ab255811	Specific staining	1/100
Astrocytes	GFAP		Chicken	Abcam	ab4674	Specific staining	1/500
			Mouse	Sigma/Merck	G3893	Specific staining	1/100
			Rabbit	Agilent	Z033429-2	Specific staining	1/100
Neurons	Neurofilament H		Chicken	Sigma/Merck	ab5539	Specific staining	1/500
	MAP2		Chicken	Abcam	ab5392	Specific staining	1/100
Secondaries	Goat anti-Chicken IgY (H+L) – 568			Life Technologies	A-11041		1/400
	Goat anti-Rabbit IgG - 647			Life Technologies	A-21245		1/400
	Donkey anti-Rat IgG – 568			Abcam	ab175475		1/400
	Donkey anti-Rabbit IgG - 568			Life Technologies	A-10042		1/400

Cell-specific antibodies highlighted in green were used for the figures presented in this study. Antibodies that only showed staining for cellular processes were not used in this study as it was difficult to identify the specific nucleus corresponding to the fluorescent signal. Markers that showed weak staining were also not included where a stronger marker for the same antibody could be used.

Supplementary Table 3 Summary of aSyn immuno-positive inclusions found by CLEM in multiple system atrophy brain donors.

Inclusion type	Donor	Brain region	Number found	Associated Figures
GCI (n=128*)	A	PUT	7 (3)	Supplementary Fig. 4a-c
	B	PUT	15 (3)	Supplementary Fig. 3a
	C	SN	64 (7)	Fig. 5a, Supplementary Fig.2
		PUT	26 (3)	Fig. 1b, Supplementary Fig.3b
	D	SN	9 (7)	Fig. 1a, Supplementary Fig. 1
	F	SN	2[$]	Fig. 3d, Supplementary Fig. 5b
	G	PUT	3[$]	Supplementary Fig. 4d-f
aSyn$^+$ dark cells (n=47*)	A	PUT	1	Fig. 5c
	C	PUT	1	Fig. 3a
	D	SN	17 (12)	Fig. 3b, Fig. 5d, Supplementary Fig. 9b-k
	F	SN	19 (10)	Fig. 3d Fig. 5c, Supplementary Fig. 10a-i
	G	PUT	3	Supplementary Fig. 10j-l
		SN	6	Fig. 4, Supplementary Figs. 9a and 16
NCI (n=20[#])	C	SN	1	Fig 2d
	D	SN	3	Fig 2a,c
	F	SN	7[#]	Fig. 2b, Fig. 5b, Supplementary Fig. 5 ([#]e)
	G	PUT	5[#]	Supplementary Fig. 7[#]
	H	SN	4	Supplementary Fig. 6
axonal inclusion (n=3)	C	SN	1	Supplementary Fig. 8a
	D	SN	1	Supplementary Fig. 8b
	F	SN	1	Supplementary Fig. 8c
Glial nuclear inclusions (n=4)	C	SN	4	Supplementary Fig. 13
Neuronal nuclear inclusions (n=2)	F	SN	1	Supplementary Fig. 5f
	H	PUT	1	Supplementary Fig. 7

GCI = glial cytoplasmic inclusion. aSyn+ = immuno-positive for aSyn. NCI = neuronal cytoplasmic inclusion. PUT = putamen. SN = substantia nigra.*not all data shown. () indicates representative subset shown in the manuscript. [#]A subset of the NCIs also contained nuclear inclusions. [$]These patients contained many more GCI's than shown here, however they weren't imaged as they didn't add any new information to this manuscript.

Supplementary Table 4 Count of gold particles and points per area for immuno-gold labelling against IBA1.

Category	1		2		3		4		5		6		7		8		9		10		Total Points	Total Gold
	P	G	P	G	P	G	P	G	P	G	P	G	P	G	P	G	P	G	P	G		
aSyn immuno-positive dark cells	33	24	99	54	366	244	188	92	93	478	410	17	100	58	153	299	76	103	94	20	1612	1389
aSyn immuno-negative dark cells	148	1080	105	83	204	1452	74	37	72	472	155	111	75	42	99	88	157	283	77	93	1166	3741
Oligodendrocyte	270	1015	157	774	126	515	134	145	76	401	225	143	153	123	181	109	259	281	217	543	1798	4049
Neuron	290	342	225	1071	133	836	130	464	161	83	214	72	322	239	733	446	625	627	497	388	3330	4568
**Totals**	**741**	**2461**	**586**	**1982**	**829**	**3047**	**526**	**738**	**402**	**1434**	**1004**	**343**	**650**	**462**	**1166**	**942**	**1117**	**1294**	**885**	**1044**		

Cells are aSyn immuno-negative unless otherwise stated. P = points counted per area. G = gold beads counted within the specified area.

Supplementary Table 5 Quantification of immuno-gold labelling against IBA1.

**Categories**	∑ Points	**Observed gold**<br>∑ Gold	**Expected gold**<br>(Total gold/Total points)*∑ points	**Chi-squared**<br>$(Obs-Exp)^2/Exp$	**% Chi-squared**<br>(Chi-squared/total Chi-squared)*100	**Relative labelling index**<br>Obs/exp	**p-value**
aSyn immuno-positive dark cells	1612	1389	2803.0	713.3	26.5	0.5	<0.05
aSyn immuno-negative dark cells	1166	3741	2027.4	1448.3	53.8	1.8	<0.05
Oligodendrocyte	1798	4049	3126.4	272.3	10.1	1.3	<0.05
Neuron	3330	4568	5790.2	258.0	9.6	0.8	<0.05
**Totals**	**7906**	**13747**		**4336.7**	**100**		
Degrees of freedom (number of categories – 1) = 7							

Cells are aSyn immuno-negative unless otherwise stated. P = points counted per area. G = gold beads counted within the specified area. The expected gold shows the amount of gold labelling which would be observed if the distribution was random. Chi-squared = category with the largest contribution to the total Chi-squared value will be more likely to contain a specific gold signal. RLI – relative labelling index. A RLI >1 shows categories which have a higher density of gold label than expected (if the labelling were random). A RLI <1 shows categories where the gold signal is less concentrated than expected. The P-value was calculated from the Chi-Square distribution reference table, with a P-value <0.05 indicating the differences between the observed and expected gold distribution are statistically significant. The shaded boxes indicate which categories meet both specificity criteria with a % Chi-squared over 10% and an RLI >1.
